# The *ytrGABCDEF* operon controls cell size, growth arrest, and lysis of distinct *Bacillus subtilis* subpopulations

**DOI:** 10.1128/spectrum.01532-26

**Published:** 2026-09-30

**Authors:** Margareth Sidarta, Luna Baruah, Pauline Hammer úr Skúoy, Ann-Britt Schäfer, Olivia Johnsson, Emma Frisk, Paula Didelot, Leo Andrekson, Michaela Wenzel

**Affiliations:** 1 Division of Molecular Bioscience, Department of Life Sciences, Chalmers University of Technology 11248, Gothenburg, Sweden; 2 Centre for Antibiotic Resistance Research in Gothenburg (CARe), Gothenburg, Sweden; Emory University School of Medicine, Atlanta, Georgia, USA

**Keywords:** cell wall homeostasis, population heterogeneity, bacterial cytological profiling, ABC transporter, *Bacillus subtilis*

## Abstract

The *Bacillus subtilis ytrGABCDEF* operon has been implicated in various cellular processes, including antibiotic and cold stress adaptation, cell wall synthesis, autolysis, sporulation, biofilm formation, motility, competence, and acetoin utilization. Yet, how these different observations fit together and the precise function of the operon remain enigmatic. In addition to its function, its topology is also uncertain. Early studies proposed a single YtrBCDEF ATP-binding cassette (ABC) transporter, while more recent studies showed evidence of two distinct ABC transporters, YtrB_2_CD and YtrE_2_F_2_. Here, we phenotypically characterized diverse *ytr* deletion mutants overexpressing or lacking the whole operon or parts thereof. Unexpectedly, we found a distinct population heterogeneity in *ytr* mutants that constitutively express either the whole operon or parts thereof. These strains displayed a substantial subpopulation of non-growing cells with measurably decreased cell length and width (“small cells”). This phenotype was most prevalent during adaptation from slow to fast growth and was exacerbated by cold. In mutants expressing an incomplete operon, small cells eventually underwent cell lysis. Taken together, our results suggest a role of the operon in controlling cell size, growth arrest, and autolysis of distinct subpopulations during growth phase transitions and temperature shifts.

**IMPORTANCE:**

*Bacillus subtilis* is a crucial bacterial model organism with considerable importance to fundamental and applied microbiology, frequently used as a model in bacterial cell biology and physiology and as a production host for various industrial applications. Despite being one of the best-characterized microbes, the *B. subtilis* genome still contains many genes and operons of unknown or poorly characterized function. The *ytrGABCDEF* operon has been the subject of a number of studies, yet neither its function nor the structure of the encoded proteins has so far been conclusively elucidated. Here, we provide new insight into the function of this operon that reconciles previous observations and suggests new directions for the further characterization of the structure-function relationship of the Ytr protein complexes.

## INTRODUCTION

The ubiquitous soil bacterium *Bacillus subtilis* is a well-characterized Gram-positive model organism, prominently used for studying cell division, cell wall synthesis, and sporulation (1–4). Cells in a *B. subtilis* population can differentiate into distinct states, including vegetative growth and endospore formation, as well as genetic competence, biofilm formation, motility, autolysis, cannibalism, and oligotrophic growth (5, 6). This breadth of possible cell types has advanced *B. subtilis* as a principal model for studying bacterial cellular differentiation (7–9).

**Fig 1:**
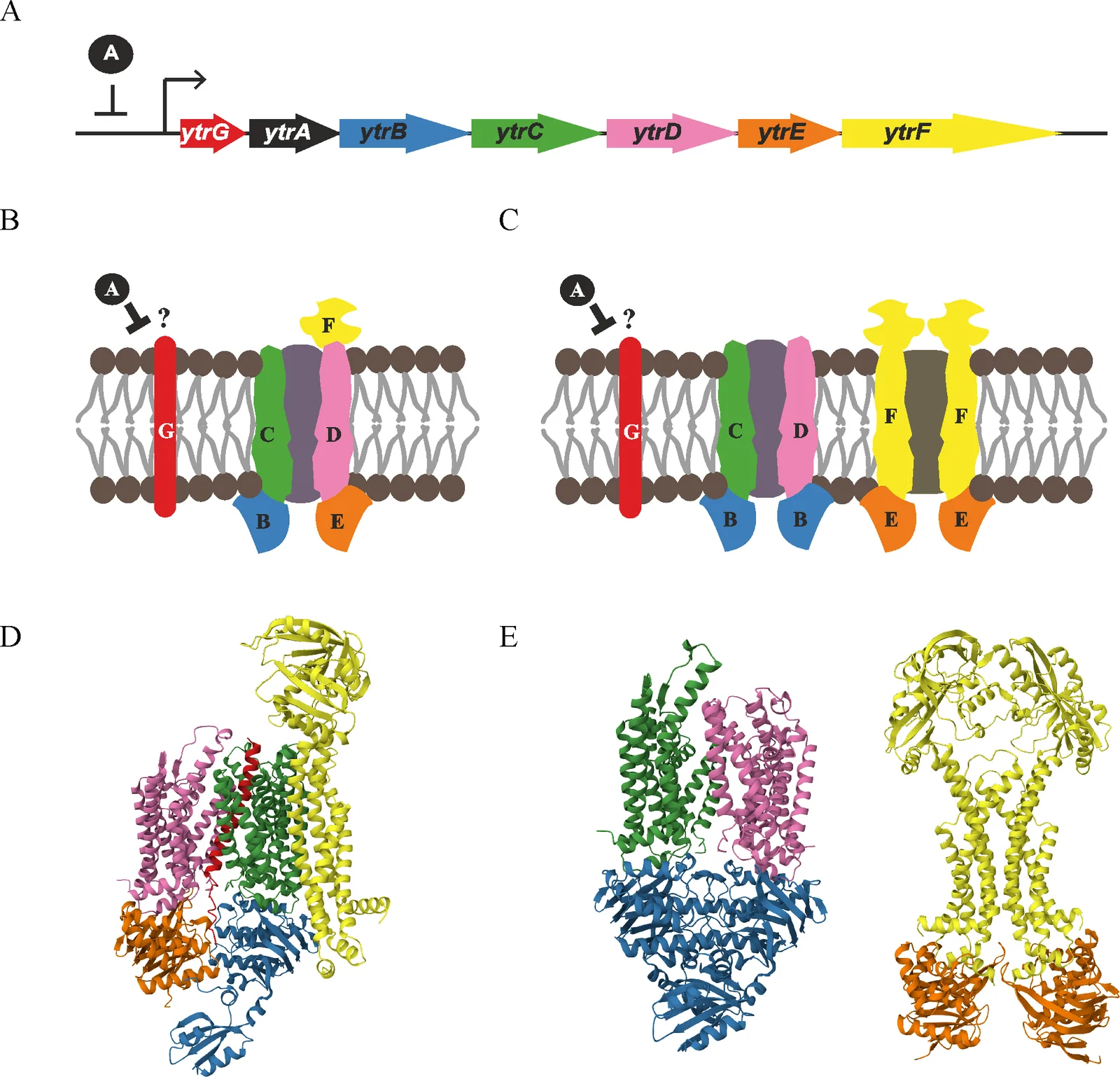
Genetic organization of the *ytr* operon and predicted subunits of the encoded ABC transporter. (**A**) Operon organization of *ytrGABCDEF*. (**B**) Schematic structure of the putative ABC transporter YtrBCDEF as suggested by Yoshida et al. (**C**) Schematic structure of the putative ABC transporters YtrB_2_CD and YtrE_2_F_2_ according to Yu et al. Note that YtrG, classified as a small transmembrane protein (18), is not included in either structural model. (**D**) Structural prediction of YtrGBCDEF according to AlphaFold3 (green: YtrC, pink: YtrD, blue: YtrB, orange: YtrE, yellow: YtrF, and red: YtrG). (**E**) Structural prediction of YtrB_2_CD and YtrE_2_F_2_ according to AlphaFold3 (green: YtrC, pink: YtrD, blue: YtrB, orange: YtrE, and yellow: YtrF). YtrG is not included in this model as AlphaFold did not provide conclusive predictions on the number of YtrG subunits for either YtrB_2_CD or YtrE_2_F_2_.

Despite having been extensively studied, the *B. subtilis* genome still contains many genes with unknown or poorly characterized functions. One such system is the *ytrGABCDEF* operon (Fig. 1A). YtrB and YtrE are ATP-binding subunits that have been proposed to, together with the transmembrane channel proteins YtrC and YtrD and the extracytoplasmic substrate-binding lipoprotein YtrF, form the putative ATP-binding cassette (ABC) transporter YtrBCDEF (Fig. 1B) (10–13). The *ytrA* gene encodes a transcriptional repressor, whose deletion results in constitutive expression of the operon (12). The small transmembrane protein YtrG possesses a single transmembrane domain that shares amino acid similarity with the first transmembrane segment of YtrF (identity 42.5%, *E* = 8^−10^) (12, 14–17). It is part of the operon but was not originally predicted to be part of the ABC transporter (12, 18).

Based on AlphaFold structural predictions, more recent studies have suggested a different transporter topology, proposing two distinct ABC transporters, YtrB_2_CD and YtrE_2_F_2_ (Fig. 1C and E) (19, 20). Although the YtrE_2_F_2_ transporter structure was indeed experimentally confirmed (20), the operon structure, which lacks a secondary promoter before the *ytrEF* genes, and near-identical transcriptomic expression patterns of the individual genes (12, 21) raise questions about how a B_2_CD and E_2_F_2_ stoichiometry could be achieved. Since the cryogenic electron microscopy structure of YtrE_2_F_2_ is derived under conditions in which only the YtrE and YtrF subunits are expressed and not the remaining genes of the operon (20), neither hypothesis has so far been proven unambiguously. Nonetheless, the YtrB_2_CD and YtrE_2_F_2_ topologies are clearly favored over the YtrBCDEF and YtrGBCDEF alternatives, which is apparent from pTM and iPTM values generated in AlphaFold3 (Fig. S1 and 2) and the predicted aligned error (PAE) visualizations generated using the PAE viewer (22) (Fig. S5 and S6), supporting the two-transporter hypothesis.

Several studies have reported a connection between the *ytr* operon and cell wall synthesis-inhibiting antibiotics, consistently finding the operon to be induced by compounds that bind to lipid-linked cell wall precursors (bactoprenol phosphate/pyrophosphate, lipid I/II) (12, 23–35). Based on these observations, it has been proposed that the *ytrGABCDEF* operon may confer resistance to such compounds (28). This hypothesis was supported by the fact that YtrF contains an N-terminal MacB-like sequence, a domain known for binding antibiotics (13, 36). However, the structural diversity of antibiotics that induce the *ytr* operon, including glycopeptides, lipopeptides, lantibiotics, defensins, and even an atypical tetracycline, challenged this hypothesis (28). Independent studies have tried and failed to establish a role for the *ytr* operon in antibiotic protection (12, 27). However, several other observations have supported a link between the *ytr* operon and cell wall synthesis and, interestingly, several cellular differentiation states of *B. subtilis* that affect and/or are affected by cell wall homeostasis (see Table S1 for a detailed summary of key observations) (11, 13, 27, 37). Thus, electron microscopy experiments revealed that the Δ*ytrA*, Δ*ytrAB*, and Δ*ytrAE* mutants display a strongly increased cell wall thickness (13). The same mutants displayed an overall increased lipid II content but reduced septal lipid II accumulation and exhibited a compromised cell wall integrity phenotype that is usually associated with an imbalance between cell wall synthesis and hydrolysis (34), confirming a role of the operon in controlling cell wall homeostasis (27).

The *ytr* operon has also been implicated in stationary phase transition, acetoin consumption, and cold shock (Table S1). Thus, expression peaks during exponential and early stationary phase and decreases during late stationary phase (11, 21). It has been suggested that the *ytr* operon is involved in the import of acetoin during transition/early stationary phase (11). Acetoin, a side product of glucose consumption, is produced and secreted in large amounts by exponentially growing *B. subtilis* cultures and is re-utilized once preferable carbon sources are exhausted (38). Since acetoin utilization is inevitably correlated with growth-phase transition, it is possible that the observed effects on acetoin uptake may be indirect. Notably, the by far strongest inducer of the *ytr* operon, apart from cell wall synthesis-inhibiting antibiotics, is cold shock (21, 39), pointing toward a role of the *ytr* operon in temperature adaptation. This was confirmed by the considerably decreased cold shock tolerance of different *ytr* mutants (27). It should be noted that cell wall autolysis in *B. subtilis* is activated by cold shock (40–42), tying these observations back into a function related to cell wall homeostasis.

Aiming to look further into the function of the operon under different temperature conditions, we assessed phenotypic changes of various deletion mutants of the *ytr* operon using bacterial cytological profiling (BCP). Surprisingly, these experiments revealed a distinct population heterogeneity in several *ytr* mutants, forming subpopulations of non-growing “small cells” characterized by reduced length and width. In mutants expressing an incomplete operon, these small cells eventually underwent lysis. This population heterogeneity, occurring most prominently during the lag-to-log-phase transition and under cold-shift conditions, corroborates the involvement of the *ytr* operon in balancing cell wall homeostasis, possibly in connection with cannibalism.

## RESULTS

In a previous study, we observed that a subset of *ytr* mutants exhibited clear growth defects and increased cell lysis at low temperatures (27). Thereby, the strongest effects were observed with strains that constitutively expressed the whole operon (Δ*ytrA*) or an incomplete ABC transporter (Δ*ytrAB*, Δ*ytrAE*, Δ*ytrABE*, Δ*ytrACD*). Single-gene deletions of transporter components had milder effects (Δ*ytrB*, Δ*ytrC*, Δ*ytrD*, Δ*ytrE*) or no effect (Δ*ytrF*), and the whole-operon mutant (Δ*ytrGABCDEF*) grew better than the wild type (27). To follow up on these observations, we used the same set of strains to further assess their growth and lysis behavior at low temperatures. To this end, we either grew cells at a constant temperature of 37 or 24°C or shifted them from 37 to 24°C upon dilution of overnight cultures. See Fig. S1 to S4 for structural and schematic representations of the different transporter mutants used in this study, taking into account both the one- and two-transporter topology hypotheses. Note that these representations are for visualization of the expressed subunits only and that the stoichiometry and structure of the transporter variants in the different mutant strains have not been experimentally confirmed.

### Population heterogeneity and “small cell” phenotype

**Fig 2:**
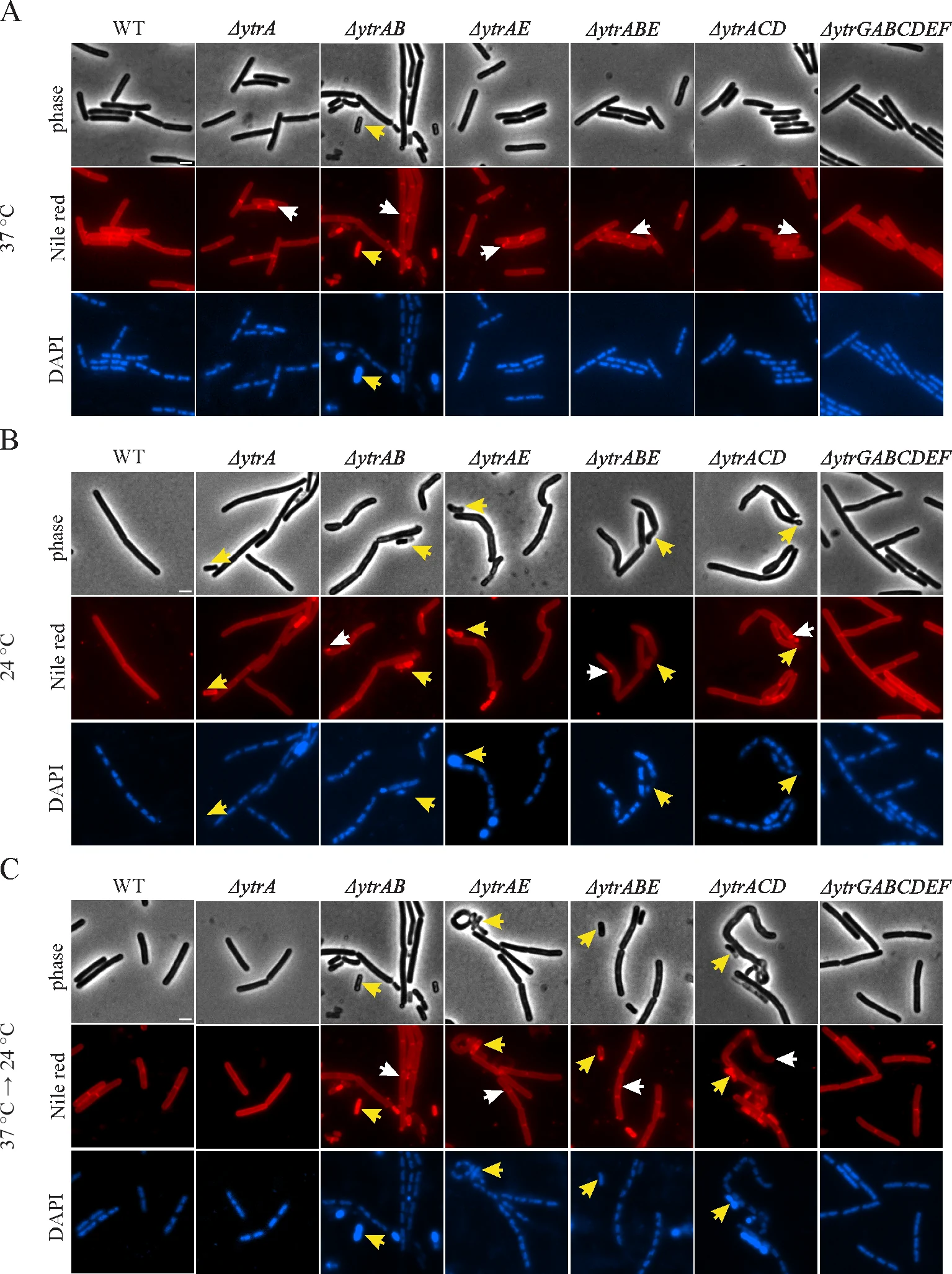
BCP of Δ*ytrA*, Δ*ytrAB*, Δ*ytrAE*, Δ*ytrABE,* Δ*ytrACD*, and Δ*ytrGABCDEF* cultures grown at constant 37°C (**A**), constant 24°C (**B**), or shifted from 37°C to 24°C after dilution of overnight cultures (**C**). Membranes were stained with Nile red and DNA with DAPI. White arrows indicate membrane foci. Yellow arrows indicate small cells. Scale bars, 2 µm. Please refer to Fig. S7 for BCP of the Δ*ytrB*, Δ*ytrC*, Δ*ytrD*, Δ*ytrE*, and Δ*ytrF* single mutants.

To gain more insight into the cold-induced lysis phenotype at the single-cell level, we performed BCP (Fig. 2; Fig. S7) (43). This method uses phase-contrast microscopy paired with the DNA dye DAPI and the membrane dye Nile red. While phase density gives insight into loss of intracellular content, the fluorescent stains aid in identifying separated cells in a chain, visualizing membrane domains and aberrant cell wall synthesis, and identifying lysing cells (44). Table S2 gives an overview of the results for all tested strains and conditions. At 37°C, the Δ*ytrAB*, Δ*ytrAE*, Δ*ytrABE*, and Δ*ytrACD* mutants showed membrane aberrations in the Nile red stain, deformed cell shapes, and subpopulations of lysing cells (Fig. 2A), correlating with their reduced fitness. At 24°C, in addition to Δ*ytrAB*, Δ*ytrAE*, Δ*ytrABE*, and Δ*ytrACD*, the Δ*ytrC* and Δ*ytrD* strains also showed membrane aberrations and cell lysis (Fig. 2B; Fig. S7). When cells were shifted from 37 to 24°C, these phenotypes became even more apparent in the same six strains (Fig. 2C; Fig. S7).

**Fig 3:**
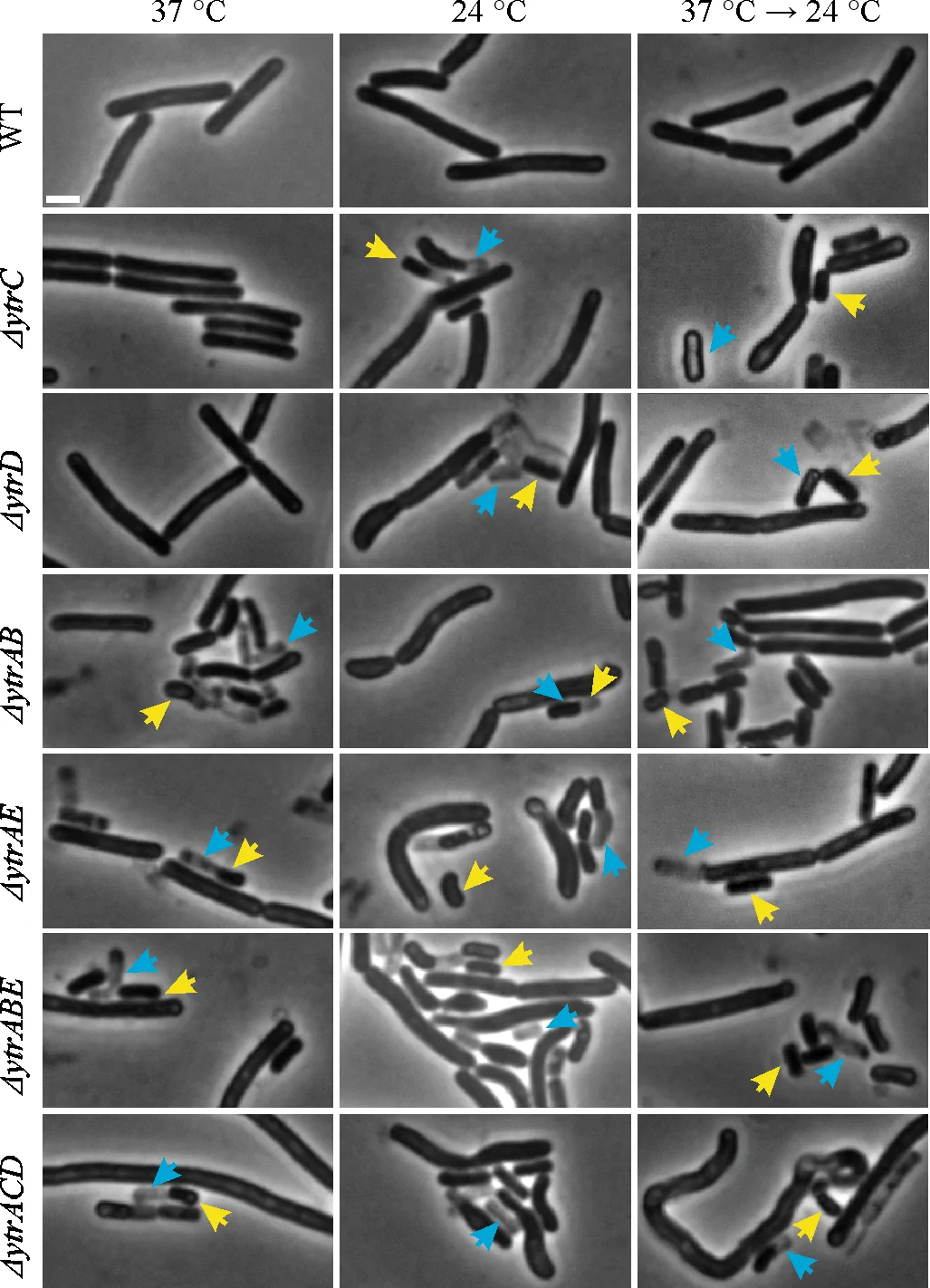
Phase-contrast microscopy of *B. subtilis* 168CA (WT) and the Δ*ytrC*, Δ*ytrD,* Δ*ytrAB*, Δ*ytrAE*, Δ*ytrABE*, and Δ*ytrACD* mutants at different temperatures. Images show log-phase cells. See Fig. S8 to S10 for images of all strains taken at different ODs. Yellow arrows indicate small cells. Blue arrows indicate lysing/lysed small cells. Scale bar, 2 µm.

Interestingly, we observed strongly heterogeneous cell populations in our BCP assays. In addition to normal cells and clearly lysed/lysing cells, we observed a “small cell” phenotype in Δ*ytrA*, Δ*ytrAB*, Δ*ytrAE*, Δ*ytrABE*, and Δ*ytrACD* under all conditions and in Δ*ytrC* and Δ*ytrD* at a constant 24°C and upon a temperature shift from 37 to 24°C. This phenotype was so distinct that we originally suspected a contamination of our cultures. After re-streaking all strains to single colonies and renewed inoculation, we noticed that the small-cell phenotype was absent from new overnight cultures. However, when the same cultures were diluted in fresh medium and grown to the early exponential growth phase again, small cells were observed in the same strains as before. These observations prompted us to check for small cells at different optical densities (ODs). Indeed, we could confirm that small cells are not prominent in the lag and stationary phase but only when cultures transition to the exponential phase. This was the case for all three temperature conditions (Fig. 3; Fig. S8 to S10).

Timelapse experiments showed that small cells originated from actively growing siblings, did not grow, and in the double and triple mutants eventually underwent lysis (Fig. S11; Videos S1 to S9). They further showed strongly increased DAPI and Nile red stains, an observation often made with lysing cells (Fig. S12). They were also not or only very weakly stained with Van-FL (labels lipid II) or Bocillin (labels penicillin-binding proteins) (Fig. S12), indicating that small cells do not actively synthesize cell wall peptidoglycan.

**Fig 4:**
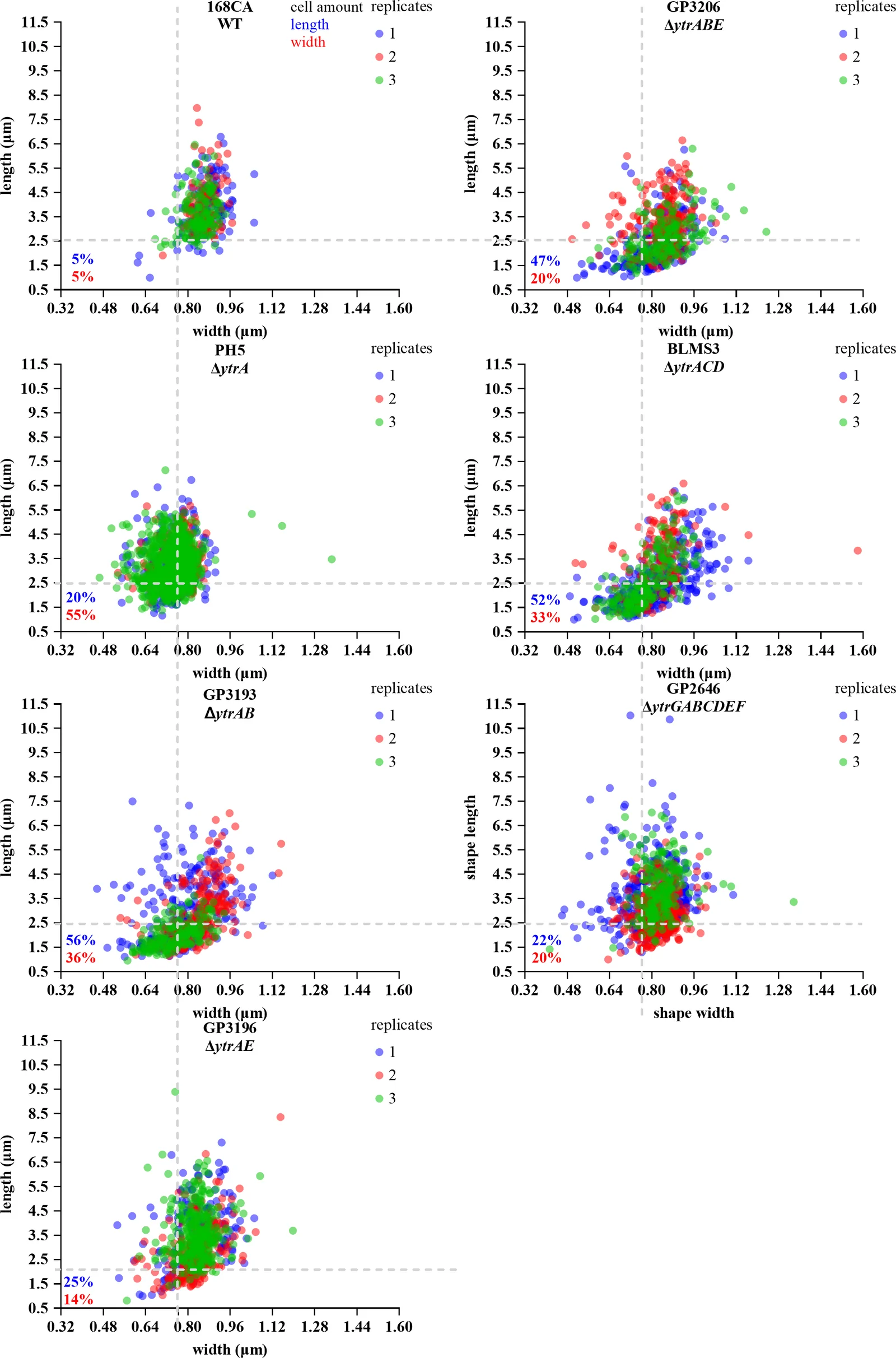
Quantification of cell length and cell width of *B. subtilis* 168CA (WT), Δ*ytrA*, Δ*ytrAB*, Δ*ytrAE*, Δ*ytrABE,* Δ*ytrACD*, and Δ*ytrGABCDEF* cell populations shifted from 37°C to 24°C after dilution of overnight cultures. Blue, red, and green data points represent three biological replicates. Dotted lines represent cutoff values for the 95th quantile for the WT (95% of the WT cell population falling above the cutoff in length and width, respectively). Percentages show the proportion of cells outside of the 5% WT cutoffs for length (red) and width (blue) calculated for pooled data sets.

To obtain quantitative information on small cells, we measured the dimensions of the strains that showed the strongest phenotypes (Δ*ytrAB*, Δ*ytrAE*, Δ*ytrABE*, and Δ*ytrACD*) and compared them to the wild type, the Δ*ytrA* strain constitutively expressing the whole operon, and the whole operon deletion Δ*ytrGABCDEF*, under temperature shift conditions where the strongest effects were observed (Fig. 4; Fig. S13). This analysis revealed that all quantified strains showed small cell populations. Thereby, Δ*ytrAB*, Δ*ytrABE*, and Δ*ytrACD* exhibited the most prominent effects in both length and width. This correlated with growth experiments, in which these three strains showed the strongest growth defects upon temperature down-shock (27). Curiously, in Δ*ytrA*, which showed shorter and thinner cells upon visual examination, yet without the strong lysis phenotype observed for the double and triple mutants (Fig. 2; Fig. S7; Table S1), 55% of the cell population fell outside the 95th WT quantile for cell width, but only 20% for length. Following up on that, we quantified the cell dimensions of this strain for all growth conditions and compared it to the WT (Fig. S14). Cell length was similar to WT at 24°C but slightly decreased at 37°C and even more so after shifting cultures from 37 to 24°C. In all conditions, Δ*ytrA* cells were considerably thinner than WT, with the mildest effects at 24°C and the strongest upon temperature shift. These observations suggest that up to 60% of the cell population cannot maintain their regular cell diameter and, to a lesser extent, length during constitutive expression of the *ytr* operon. Yet, excessive small cell formation, cell deformation, and lysis only set in when incomplete transporter variants are constitutively expressed, in particular Δ*ytrAB*, Δ*ytrABE*, and Δ*ytrACD*. Assuming the two-transporter topology is correct, this could point toward a possible function of YtrB_2_CD in preventing cell lysis during incorporation of new lateral cell wall material.

It has previously been speculated that the *ytr* operon may possibly control autolysin activity due to sequence homology of the C-terminus of YtrF with FtsX (13). In *B. subtilis*, the FtsEX complex interacts with and activates the cell wall hydrolase CwlO (45). It is therefore conceivable that the lysis phenotype observed in our *ytr* mutants is caused by autolysis, likely due to an imbalance of cell wall turnover. To assess this, we employed an autolysin mutant lacking six autolysins (strain KS19, Δ*lytABC::kan* Δ*lytD::tet* Δ*lytF::spc* Δ*lytE::cat*), which is insensitive to both cold- and antibiotic-induced autolysis (27, 46). We transformed the Δ*ytrA* (strain AS2) and Δ*ytrABE* (strain AS3) deletions into the KS19 strain background and performed BCP to assess the occurrence and lysis of small cells (Fig. S15 to S18). As expected, AS3 still formed small cells, yet without cell lysis (Fig. S15 and S18), confirming that it is indeed autolytic enzyme activity that causes the small cell lysis phenotype. This was also confirmed by timelapse microscopy (Video S10). Interestingly, KS19 also showed non-lysing small cells, yet to a much lesser extent than AS3 (Fig. S15 and S16), suggesting that the impairment of autolysin activity induces the small-cell phenotype independently of the *ytr* operon. Strikingly, small cells were exceedingly rare in AS2 (Fig. S15 and S17), even compared to KS19, implying that overexpression of the complete functional *ytr* operon mitigates this effect. AS2 also showed slimmer cells (Fig. S15 and S17), as observed in its donor strain PH5, despite the propensity of the parent strain KS19 to form thick, bulging cells (Fig. S15 and S16). This suggests that the overexpression of the full *ytr* operon also reduces the cell wall strain caused by continued cell wall synthesis in the absence of LytA-F.

## DISCUSSION

In this study, we identified the *ytrGABCDEF* operon as a factor in bacterial cell size control during growth phase adaptations and cold stress and showed that its dysregulation leads to a pronounced small-cell phenotype in distinct subpopulations.

Constitutive expression of the whole operon (Δ*ytrA*) led to large subpopulations with reduced cell width and length, yet without pronounced cell lysis, death, or shape deformations. This observation suggests that the *ytr* operon controls cell size, whereby width was more affected than length. No cell division impairment was observed, suggesting that the operon may exert its activity by controlling lateral cell wall expansion. This may occur either by regulating the activity of the elongasome, the cell wall synthesis complex that incorporates new peptidoglycan building blocks into the existing cell wall structure, or by modulating the activity of autolysins, which cleave the peptidoglycan layer to allow incorporation of new cell wall material during cell growth. This is in line with the observation that Δ*ytrA* possesses a considerably thicker cell wall than WT (13) and showed stronger Van-FL labeling, indicative of more free lipid II in the cell membrane (27). These observations could be due to an imbalance between cell wall synthesis and autolysis, leading to a build-up of additional peptidoglycan layers.

Constitutive expression of an incomplete operon (Δ*ytrAB*, Δ*ytrAE*, Δ*ytrABE*, Δ*ytrACD*) led to cell shape deformations and cell lysis, which was strongly exacerbated by cold, a known trigger of autolysis in *B. subtilis* (40–42). While these mutants were strongly growth-impaired and more sensitive to cold-induced autolysis, Δ*ytrA*, while reaching lower cell densities than WT, grew better than the double mutants and lysed more slowly than WT under prolonged cold stress (27). Similarly, in our microscopy experiments, Δ*ytrA* populations did not show excessive cell lysis while the small cell populations were lysis-prone in the double and triple mutants (Fig. S11; Videos S1 to S9). Since Δ*ytrAB* and Δ*ytrAE* also displayed an increased cell wall thickness comparable to Δ*ytrA* (13), a thicker peptidoglycan layer cannot be the decisive factor underlying this difference, suggesting a key role of autolysins in the small cell lysis phenotype. This was indeed confirmed by the absence of small cell lysis in a strain background lacking six autolytic enzymes (Fig. S15 to S18).

Interestingly, the whole-operon deletion strain displayed no pronounced phenotypic deviation from the WT in almost all tested assays, the only exception being reduced or delayed sporulation and reduced Van-FL labeling (11, 13, 27). Here, we observed no lysis or cell shape deformations but could measure a slight shift toward a small-cell subpopulation. In that sense, the whole operon deletion resembles the Δ*ytrA* deletion strain, yet to a much lesser extent. Similarly, the whole-operon deletion strain showed a slightly thicker cell wall than WT, but not as pronounced as the Δ*ytrA*, Δ*ytrAB*, and Δ*ytrAE* strains (13). The observation that the absence of the operon elicits similar, if milder, phenotypes as its constitutive expression, while the constitutive expression of a partial operon results in detrimental phenotypic defects, could point toward a system with two complementary components. This notion could support the two-transporter topology proposed by Mahendran et al. (19) and Yu et al. (20). If the YtrB_2_CD and YtrE_2_F_2_ complexes work in concert with each other, an imbalance due to impairment of one of the two systems could cause more detrimental effects than the equal overexpression or absence of both. Another observation in support of two distinct transmembrane complexes is the stronger phenotype of the Δ*ytrAB*, Δ*ytrABE*, and Δ*ytrACD* mutants compared to the Δ*ytrAE* strain, suggesting that impairment of YtrB_2_CD elicits stronger effects in terms of lysis and cell shape deformations than impairment of YtrE_2_F_2_. However, these new hypotheses remain speculative and will have to be tested in further studies.

Our observation that constitutive expression of an incomplete operon causes cell shape deformations and lysis could place the *ytr* operon into the context of cannibalism. In this scenario, lysing cells will serve as food sources for the rest of the population. Yu et al. observed that constitutive expression of the *ytr* operon resulted in a “small puncta” colony morphology (20). This phenotype occurred after prolonged (>2 weeks) incubation of plates and was characterized by the disappearance of the original colony mass and concomitant occurrence of small microcolonies (puncta) growing on the original colony area, indicating that surviving siblings grow on top of their lysed relatives. This colony morphology is perfectly in line with the small-cell and lysis phenotypes observed here. Yu et al. could pinpoint this pattern to overexpression of *ytrEF,* while overexpression of *ytrBCD* did not result in the same phenotype (20). These findings are consistent with (i) a function of YtrE_2_F_2_ in regulating autolysis and (ii) distinct functions of YtrB_2_CD and YtrE_2_F_2_ (20). It also matches the growth phenotypes of the whole-operon mutant, which generally grows better than the WT, entering stationary phase later and reaching higher end ODs, and overcomes cold shifts faster. All these observations tie in with a lack of an autolysis-stimulating factor.

However, in our hands, Δ*ytrA* seemed less prone to cell lysis. Thus, despite also lysing, Δ*ytrA* cultures retained higher cell densities than WT upon prolonged incubation at 4°C in our previous study (27). Here, Δ*ytrA* cultures were not prone to lysis in microscopy experiments, in contrast to the double and triple mutant strains. It is conceivable that an intact *ytr* operon stimulates the emergence of a more resilient subpopulation of cells, characterized by decreased cell size but not by increased cell lysis and thus distinct from the small-cell phenotype observed in the diverse deletion mutants. Small cell size is a known adaptation to survive low-nutrient conditions (6). *B. subtilis* is known to lyse during stationary phase, releasing nutrients to promote prolonged growth of sibling cells to delay sporulation (47). Taking together our observations here and in previous studies (13, 27) with the colony morphology observed by Yu et al. (20), we propose that the *ytr* operon may be responsible for the formation of lysis-resistant subpopulations and stimulates cell lysis of the remaining population. We speculate that this may be achieved by concerted action of the *ytrBCD* and *ytrEF* genes, likely forming two distinct transmembrane complexes of YtrB_2_CD and YtrE_2_F_2_ topology. It can be further speculated that YtrE_2_F_2_ could enhance lysis of non-resistant subpopulations, while YtrB_2_CD could possibly stabilize cannibalistic cells against lytic action. This could be achieved either by modulation of autolysin or elongasome activity, or even a combination of both. Whether such modulation may be due to a direct interaction of the Ytr proteins with any component of the autolytic and/or elongasome machineries remains to be explored in future studies.

It should be noted that none of the suggested transporter topologies account for the small monotopic transmembrane protein YtrG. However, we could show that YtrG contributes considerably to the small-cell phenotype, the Δ*ytrG* strain being the only *ytr* single mutant that showed a pronounced small-cell phenotype, on par with that of the double mutants (48). This observation suggests that YtrG plays an important role in the function of the *ytr* operon that has so far remained unexplored and should be addressed in future studies.

Another unresolved question is how population heterogeneity between normal cells and small cells is established. One possibility is genetic bimodality, which is generally subject to an activation threshold determined by stochastic variation in the concentration of a transcriptional activator, for example, phosphorylated Spo0A in the case of competence, sporulation, and biofilm formation (49, 50). The basis for this differential activation is the ability of a promoter to respond to different activator concentrations (49, 50). Salzberg et al. found that an increase in YtrA concentration caused an upshift in electromobility shift assays, suggesting the possibility of multiple promoter binding sites (12). Such a concentration-dependent shift in promoter binding could potentially underlie a bimodal regulation of the *ytr* operon. Furthermore, it has been postulated that reduced cell size due to slower growth rate influences the bimodal control of motility (49, 51). Given our observation of the small-cell phenotype, a similar concentration effect due to reduced cell volume could play a role in the effects of the *ytr* operon. Since the *ytr* operon is induced during growth phase transitions, cold shock, and cell wall biosynthesis inhibition, all conditions under which the equilibrium between cell wall synthesis and autolysis is shifting, it can further be speculated that imbalanced cell wall turnover may contribute to deciding cell fate.

## MATERIAL AND METHODS

### Bioinformatics

All *B. subtilis* gene sequences were obtained from the SubtiWiki database (18). Structural predictions of protein complexes were generated in AlphaFold3 version 2 (DeepMind) (52). Predictions were performed locally using the official AlphaFold3 implementation with model weights provided under the academic license. For each complex, five random seeds were evaluated, generating five structural samples per seed. The most likely structure proposed by the AlphaFold3 internal ranking system was selected for downstream analyses. All analyses were performed using default recommended settings, including automatic multiple sequence alignment generation.

### Strains and growth conditions

Strains and plasmids used in this study are listed in Table S3. Unless otherwise stated, all cultures were grown in lysogeny broth (LB). Experiments were performed at constant 37 or 24°C, or after a temperature shift from 37 to 24°C. Cultures were kept under continuous agitation to ensure optimal oxygen supply. *B. subtilis* strains were transformed with chromosomal DNA using a standard starvation protocol (53). All transformed strains were confirmed by PCR.

### BCP

BCP was performed as described in Schäfer et al. (44). In short, *B. subtilis* strains were grown to an OD_600_ of 0.3 and subsequently stained with 0.5 μg/mL Nile red (Invitrogen, membrane stain) and 1 μg/mL DAPI (Invitrogen, DNA stain) for 5 min. Van-FL and Bocillin stains were performed as described previously (27). All microscopy samples (0.5 µL) were spotted on glass slides coated with a thin film of 1.2% agarose and covered with a poly-L-dopamine-coated coverslip as described previously (54). Samples were imaged on a Nikon Eclipse Ti2 inverted fluorescence microscope equipped with a CFI Plan Apochromat objective (DM Lambda 100X Oil N.A. 1.45, W.D. 0.13 mm, Ph3), a Lumencor Sola SE II FISH 365 light source, a Photometrics PRIME BSI camera, an Okolab incubator, and Nis-Elements AR 5.21.03 software.

### Phase-contrast microscopy

For assessment of small cells at different ODs, strains were either grown at constant 24 or 37°C or shifted from 37 to 24°C upon dilution of overnight cultures. Samples were taken at an OD_600_ of 0.1, 0.4, and 2, spotted on agarose-covered glass slides, and examined by phase-contrast microscopy.

### Timelapse microscopy

Overnight cultures grown at 37°C were diluted in fresh medium to a final OD_600_ of 0.05 and grown in liquid culture at 24°C for 4 h. Cultures were then re-adjusted to an OD_600_ of 0.05 and subsequently spotted on timelapse slides. Timelapse slides were prepared by placing a small (~5 × 5 mm) film of 1.2% agarose/10% LB into a 25 µL gene frame (Thermo Scientific, AB0576) glued to a microscopy glass slide. The remaining free gene frame area was carefully filled with LB to avoid shrinking of the agarose patch due to dehydration. After cell suspensions (0.5 µL) were spotted, samples were sealed with a glass coverslip. Timelapses were recorded at 24°C by taking phase-contrast images every 5 min for 6 h.

### Image analysis and quantification

All microscopy images were processed and analyzed with Fiji (55). Small cells were quantified based on cell length and width using Fiji and the ImageJ plugin MicrobeJ (55, 56). The MicrobeJ parameters for bacterial detection in phase contrast were set to default segmentation with an area of 0.4 μm^2^-max. All other parameters remained at default settings. All chained cells were assessed and separated using the MicrobeJ manual editing interface (56). The 95th WT quantiles of cell length and width were determined by sorting the respective data tables. All cells with values outside of the 95th WT quantiles, either in length or width, were categorized as small cells. Distribution plots were generated and processed in GraphPad Prism version 10.6.1.

## Data Availability

All data, metadata, and methods used to reach the conclusions of this study are included in the main text and Supplemental Information. Raw image files will be made available upon request.

## SUPPLEMENTAL MATERIAL

Supplemental tables and figures spectrum.01532-26-s0001.pdf Tables S1 to S3 and Fig. S1 to S18.

Video S1 spectrum.01532-26-s0002.avi Timelapse microscopy of Bacillus subtilis 168 CA (WT). Cultures were adjusted to an OD600 of 0.05 before spotting onto timelapse slides. Images were recorded every 5 min for 6 h. Field of view, 134.5 µm.

Video S2 spectrum.01532-26-s0003.avi Timelapse microscopy of Bacillus subtilis PH5 (ΔytrA). Cultures were adjusted to an OD600 of 0.05 before spotting onto timelapse slides. Images were recorded every 5 min for 6 h. Field of view, 134.5 µm.

Video S3 spectrum.01532-26-s0004.avi Timelapse microscopy of Bacillus subtilis GP3193 (ΔytrAB). Cultures were adjusted to an OD600 of 0.05 before spotting onto timelapse slides. Images were recorded every 5 min for 6 h. Field of view, 134.5 µm.

Video S4 spectrum.01532-26-s0005.avi Timelapse microscopy of Bacillus subtilis GP3196 (ΔytrAE). Cultures were adjusted to an OD600 of 0.05 before spotting onto timelapse slides. Images were recorded every 5 min for 6 h. Field of view, 134.5 µm.

Video S5 spectrum.01532-26-s0006.avi Timelapse microscopy of Bacillus subtilis GP3206 (ΔytrABE). Cultures were adjusted to an OD600 of 0.05 before spotting onto timelapse slides. Images were recorded every 5 min for 6 h. Field of view, 134.5 µm.

Video S6 spectrum.01532-26-s0007.avi Timelapse microscopy of Bacillus subtilis BLMS3 (ΔytrACD). Cultures were adjusted to an OD600 of 0.05 before spotting onto timelapse slides. Images were recorded every 5 min for 6 h. Field of view, 134.5 µm.

Video S7 spectrum.01532-26-s0008.avi Timelapse microscopy of Bacillus subtilis GP2646 (ΔytrGABCDEF). Cultures were adjusted to an OD600 of 0.05 before spotting onto timelapse slides. Images were recorded every 5 min for 6 h. Field of view, 134.5 µm.

Video S8 spectrum.01532-26-s0009.avi Timelapse microscopy of Bacillus subtilis PD3 (ΔytrC). Cultures were adjusted to an OD600 of 0.05 before spotting onto timelapse slides. Images were recorded every 5 min for 6 h. Field of view, 134.5 µm.

Video S9 spectrum.01532-26-s0010.avi Timelapse microscopy of Bacillus subtilis PD2 (ΔytrC). Cultures were adjusted to an OD600 of 0.05 before spotting onto timelapse slides. Images were recorded every 5 min for 6 h. Field of view, 134.5 µm.

Video S10 spectrum.01532-26-s0011.mp4 Timelapse microscopy of Bacillus subtilis AS3 (ΔlytABC::kan ΔlytD::tet ΔlytF::spc ΔlytE::cat ΔytrA::ery ΔytrB ΔytrE). Cultures were adjusted to an OD600 of 0.05 before spotting onto timelapse slides. Images were recorded every 5 min for 6 h. Field of view, 134.5 µm. Note that KS19 and derivatives are impaired in autolytic cell separation, causing excessive chain formation.

ASM does not own the copyrights to Supplemental Material that may be linked to, or accessed through, an article. The authors have granted ASM a non-exclusive, world-wide license to publish the Supplemental Material files. Please contact the corresponding author directly for reuse.
