# Supplementary material for: The ytrGABCDEF operon controls cell size, growth arrest, and lysis of distinct Bacillus subtilis subpopulations
Source: Microbiol Spectr. 2026 Sep 30;14(11):e01532-26. doi: 10.1128/spectrum.01532-26 (PMC13729185; doi:10.1128/spectrum.01532-26)
Supplement: Supplementary file 1 [file spectrum.01532-26-s0001.pdf]

Supplementary Information

The *ytrGABCDEF* operon controls cell size, growth arrest, and lysis of distinct *Bacillus subtilis* subpopulations

Margareth Sidarta[1,2], Luna Baruah[1,2], Pauline Hammer úr Skúoy[1], Ann-Britt Schäfer[1,2], Olivia Johnsson[1], Emma Frisk[1], Paula Didelot[1], Leo Andrekson[1,2], Michaela Wenzel[1,2]*

[1]Division of Molecular Bioscience, Department of Life Sciences, Chalmers University of Technology, Gothenburg, Sweden

[2]Centre for Antibiotic Resistance Research in Gothenburg (CARe), Gothenburg, Sweden

*Corresponding author: Michaela Wenzel (wenzelm@chalmers.se)

Supplementary tables

Table S1: Key observations from previous studies related to the *ytrGABCDEF* operon.
Table S2: Results summary of bacterial cytological profiling experiments shown in **Figure 2** and **S7**.
Table S3: Strains used in this work.

Supplementary figures

Figure S1: AlphaFold3 structural predictions of transporter structure in the different deletion mutants used in this study assuming a single transporter complex.
Figure S2: AlphaFold3 structural predictions of transporter structure in the different deletion mutants used in this study assuming two distinct transporters, $YtrB_2CD$ and $YtrE_2F_2$.
Figure S3: Schematic structure of the putative ABC transporter encoded by the *ytrGABCDEF* operon (WT) and transporter expression in the different deletion mutants used in this study assuming a single transporter complex.
Figure S4: Schematic structure of the putative ABC transporter encoded by the *ytrGABCDEF* operon (WT) and transporter expression in the different deletion mutants used in this study assuming two distinct transporters, $YtrB_2CD$ and $YtrE_2F_2$.
Figure S5: Predicted aligned error (PAE) visualizations of the YtrBCDEF (**A**) and YtrGBCDEF (**B**) transporter topologies.
Figure S6: Predicted aligned error (PAE) visualizations of the $YtrB_2CD$ (**A**) and $YtrE_2F_2$ (**B**) transporter topologies.
Figure S7: Bacterial cytological profiling (BCP) of WT, Δ*ytrB*, Δ*ytrC*, Δ*ytrD*, Δ*ytrE,* and Δ*ytrF* cultures grown at constant 37 °C (**A**), constant 24 °C (**B**), or shifted from 37 °C to 24 °C after dilution of overnight cultures (**C**).
Figure S8: Phase contrast microscopy of *B. subtilis* 168CA and *ytr* deletion mutants grown at 37 °C.
Figure S9: Phase contrast microscopy of *B. subtilis* 168CA and *ytr* deletion mutants grown at 24 °C.
Figure S10: Phase contrast microscopy of *B. subtilis* 168CA and *ytr* deletion mutants shifted from 37 to 24 °C after dilution of overnight cultures.
Figure S11: Exemplary timelapse images of small cells.
Figure S12: Different fluorescent stainings of small cells and normal cells.
Figure S13: Scatter plots of cell width and length in different *ytr* deletion mutants.
Figure S14: Quantification of cell length and width of *B. subtilis* 168CA (WT) and PH5 (Δ*ytrA*) at different temperature conditions.
Figure S15: Bacterial cytological profiling of the *B. subtilis* autolysin mutants KS19 (*trpC2* Δ*lytABC::kan* Δ*lytD::tet* Δ*lytF::spc* Δ*lytE::cat*), AS2 (*trpC2* Δ*lytABC::kan* Δ*lytD::tet* Δ*lytF::spc* Δ*lytE::cat* Δ*ytrA::ery*), and AS3 (*trpC2* Δ*lytABC::kan* Δ*lytD::tet* Δ*lytF::spc* Δ*lytE::cat* Δ*ytrA::ery* Δ*ytrB* Δ*ytrE*).
Figure S16: Overview image of bacterial cytological profiling of *B. subtilis* KS19 (*trpC2* Δ*lytABC::kan* Δ*lytD::tet* Δ*lytF::spc* Δ*lytE::cat*) corresponding to **Figure S15**.
Figure S17: Overview image of bacterial cytological profiling of *B. subtilis* AS2 (*trpC2* Δ*lytABC::kan* Δ*lytD::tet* Δ*lytF::spc* Δ*lytE::cat* Δ*ytrA::ery*) corresponding to **Figure S15**.
Figure S18: Overview image of bacterial cytological profiling of *B. subtilis* AS3 (*trpC2* Δ*lytABC::kan* Δ*lytD::tet* Δ*lytF::spc* Δ*lytE::cat* Δ*ytrA::ery* Δ*ytrB* Δ*ytrE*) corresponding to **Figure S15**.

Supplementary Video legends

Video S1: Timelapse microscopy of *B. subtilis* 168 CA (WT).
Video S2: Timelapse microscopy of *B. subtilis* PH5 (Δ*ytrA*).
Video S3: Timelapse microscopy of *B. subtilis* GP3193 (Δ*ytrAB*).
Video S4: Timelapse microscopy of *B. subtilis* GP3196 (Δ*ytrAE*).

Video S5: Timelapse microscopy of *B. subtilis* GP3206 (Δ*ytrABE*).
Video S6: Timelapse microscopy of *B. subtilis* BLMS3 (Δ*ytrACD*).
Video S7: Timelapse microscopy of *B. subtilis* GP2646 (Δ*ytrGABCDEF*).
Video S8: Timelapse microscopy of *B. subtilis* PD3 (Δ*ytrC*).
Video S9: Timelapse microscopy of *B. subtilis* PD2 (Δ*ytrD*).
Video S10: Timelapse microscopy of *B. subtilis* AS3 (Δ*lytABC::kan* Δ*lytD::tet* Δ*lytF::spc* Δ*lytE::cat* Δ*ytrA::ery* Δ*ytrB* Δ*ytrE*)

Supplementary references

Table S1: Key observations from previous studies related to the *ytrGABCDEF* operon.

Category	Key observations	References
**structure**	- prediction of YtrBCDEF as ABC transporter, YtrA as repressor, YtrG function unknown<br>- AlphaFold prediction of $YtrB_2CD$ and $YtrE_2F_2$ as two distinct ABC transporters<br>- $YtrE_2F_2$ transporter structure was confirmed by cryo-EM	[1–4]<br>[5]<br>[6]
**growth phase**	- expressed during early stationary phase in minimal medium<br>- expressed in transition phase in M9 medium	[2]<br>[7]
**cold shock**	- highly expressed during cold shock<br>- Δ*ytrA* displayed reduced fitness at 16 °C<br>- deletion mutants showed varying degrees of growth defects during cold shock<br>- constitutive expression of the *ytr* operon may promote cold-induced autolysis	[7,8]<br>[9]<br>[10]<br>[10]
**acetoin utilization**	- inactivation of the operon resulted in slower acetoin consumption<br>- acetoin does not act as inducer of the operon	[2]<br>[2]
**sporulation**	- inactivation of *ytrF* or *ytrGABCDEF* led to lower sporulation efficiency<br>- Δ*ytrA* showed lower sporulation efficiency<br>- Δ*ytrGABCDEF* showed delayed sporulation<br>- similar sporulation efficiency of wild type, Δ*ytrA,* and Δ*ytrGABCDEF* was observed after 24 h	[2]<br>[9]<br>[10]<br>[10]
**genetic competence**	- Δ*ytrA* and other deletion mutants showed reduced competence<br>- Δ*ytrGABCDEF,* Δ*ytrF, and Pxyl-ytrF* showed normal competence	[4,9]<br>[4]
**biofilm**	- Δ*ytrA* showed altered biofilm morphology<br>- Δ*ytrGABCDEF* exhibited normal biofilm morphology	[4]
**motility**	- constitutive expression of an incomplete operon alters colony morphology indicative of reduced swarming motility<br>- constitutive expression of the operon alters colony morphology indicative of reduced swarming motility	[10]<br>[6]
**cell wall biosynthesis (CWB)**	- Δ*ytrA,* Δ*ytrAB,* and Δ*ytrAE* strains possess a thicker cell wall than WT and Δ*ytrGABCDEF*<br>- induced by antibiotics that inhibit membrane-bound steps of the lipid II/bactoprenol cycle<br>- Δ*ytrA* showed increased nisin resistance in acute shock experiments<br>- increased nisin resistance could not be reproduced<br>- a role of the operon role in antibiotic adaptation could not be established, but its relevance for CWB was confirmed<br>- constitutive expression of the whole operon or of *ytrEF* leads to 'small puncta' colony morphology after prolonged growth	[4]<br>[3,11–20]<br>[21]<br>[10]<br>[10]<br>[6]
**other**	- lowest expression under diamide and ethanol treatment<br>- higher fitness of deletion mutants in glucose/malate/NH_4 and malate/NH_4	[7]<br>[9]

Table S2: Qualitative results summary of bacterial cytological profiling experiments shown in **Figure 2** and **S7**. Small cells were defined by both reduced length and width (see **Figure 4**) as well as intense Nile red and DAPI signals (see **Figure S12**). Lysed cells were defined by loss of phase contrast. Any deviation from regular rod shape was defined as 'deformed'. Clearly distinct Nile red foci were defined as 'membrane foci'. Phenotypes were counted as present when they were clearly identifiable from microscopy images. For quantitative information on the proportions and measurements of small cells under relevant conditions, see **Figure 4** and **S13**.

Strain	Nile red	DAPI	phase contrast
	37 ºC		
168CA (WT)	smooth	normal	normal
PH5 (Δ*ytrA)*	membrane foci	normal	small cells
PH1 (Δ*ytrB)*	smooth	normal	normal
PD3 (Δ*ytrC)*	membrane foci	normal	normal
PD2 (Δ*ytrD)*	membrane foci	normal	normal
PH2 (Δ*ytrE)*	smooth	normal	normal
PD1 (Δ*ytrF)*	smooth	normal	normal
GP3193 (Δ*ytrAB)*	membrane foci*	normal*	deformed, small cells, lysed cells
GP3196 (Δ*ytrAE)*	membrane foci*	normal*	deformed, small cells, lysed cells
GP3206 (Δ*ytrABE)*	membrane foci*	normal*	deformed, small cells, lysed cells
BLMS3 (Δ*ytrACD)*	membrane foci*	normal*	deformed, small cells, lysed cells
GP2646 (Δ*ytrGABCDEF)*	smooth	normal	normal
	24 ºC		
168CA (WT)	smooth	normal	normal
PH5 (Δ*ytrA)*	smooth	normal	small cells
PH1 (Δ*ytrB)*	smooth	normal	normal
PD3 (Δ*ytrC)*	membrane foci*	normal*	deformed, small cells, lysed cells
PD2 (Δ*ytrD)*	membrane foci*	normal*	deformed, small cells, lysed cells
PH2 (Δ*ytrE)*	smooth	normal	normal
PD1 (Δ*ytrF)*	smooth	normal	normal
GP3193 (Δ*ytrAB)*	membrane foci*	normal*	deformed, small cells, lysed cells
GP3196 (Δ*ytrAE)*	membrane foci*	normal*	deformed, small cells, lysed cells
GP3206 (Δ*ytrABE)*	membrane foci*	normal*	deformed, small cells, lysed cells
BLMS3 (Δ*ytrACD)*	membrane foci*	normal*	deformed, small cells, lysed cells
GP2646 (Δ*ytrGABCDEF)*	smooth	normal	normal
	37 ºC → 24 ºC		
168CA (WT)	smooth	normal	normal
PH5 (Δ*ytrA)*	smooth	normal	small cells
PH1 (Δ*ytrB)*	smooth	normal	normal
PD3 (Δ*ytrC)*	membrane foci*	normal*	deformed, small cells, lysed cells
PD2 (Δ*ytrD)*	membrane foci*	normal*	deformed, small cells, lysed cells
PH2 (Δ*ytrE)*	smooth	normal	normal
PD1 (Δ*ytrF)*	smooth	normal	normal
GP3193 (Δ*ytrAB)*	membrane foci*	normal*	deformed, small cells, lysed cells
GP3196 (Δ*ytrAE)*	membrane foci*	normal*	deformed, small cells, lysed cells
GP3206 (Δ*ytrABE)*	membrane foci*	normal*	deformed, small cells, lysed cells
BLMS3 (Δ*ytrACD)*	membrane foci*	normal*	deformed, small cells, lysed cells
GP2646 (Δ*ytrGABCDEF)*	smooth	normal	normal

*Small cells show aberrant and extraordinarily bright membrane and DNA stains.

Table S3: Strains used in this work.

strain	genotype	origin
B. subtilis 168CA	*trpC2*	[22]
B. subtilis AS2	*trpC2 ΔlytABC::kan ΔlytD::tet ΔlytF::spc ΔlytE::cat ΔytrA::ery*	PH5 → KS19
B. subtilis AS3	*trpC2 ΔlytABC::kan ΔlytD::tet ΔlytF::spc ΔlytE::cat ΔytrA::ery ΔytrB ΔytrE*	GP3206 → KS19
B. subtilis BLMS3	*trpC2 ΔytrA::ery ΔytrCD*	[4]
B. subtilis GP2646	*trpC2 ΔytrGABCDEF::ery*	[4]
B. subtilis GP3193	*trpC2 ΔytrA::ery ΔytrB*	[4]
B. subtilis GP3196	*trpC2 ΔytrA::ery ΔytrE*	[4]
B. subtilis GP3206	*trpC2 ΔytrA::ery ΔytrB ΔytrE*	[4]
B. subtilis KS19	*trpC2 ΔlytABC::kan ΔlytD::tet ΔlytF::spc ΔlytE::cat*	[23]
B. subtilis PD1	*trpC2 ΔytrF::kan*	[10]
B. subtilis PD2	*trpC2 ΔytrD::kan*	[10]
B. subtilis PD3	*trpC2 ΔytrC::kan*	[10]
B. subtilis PH1	*trpC2 ΔytrB::ery*	[10]
B. subtilis PH2	*trpC2 ΔytrE::ery*	[10]
B. subtilis PH5	*trpC2 ΔytrA::ery*	[10]

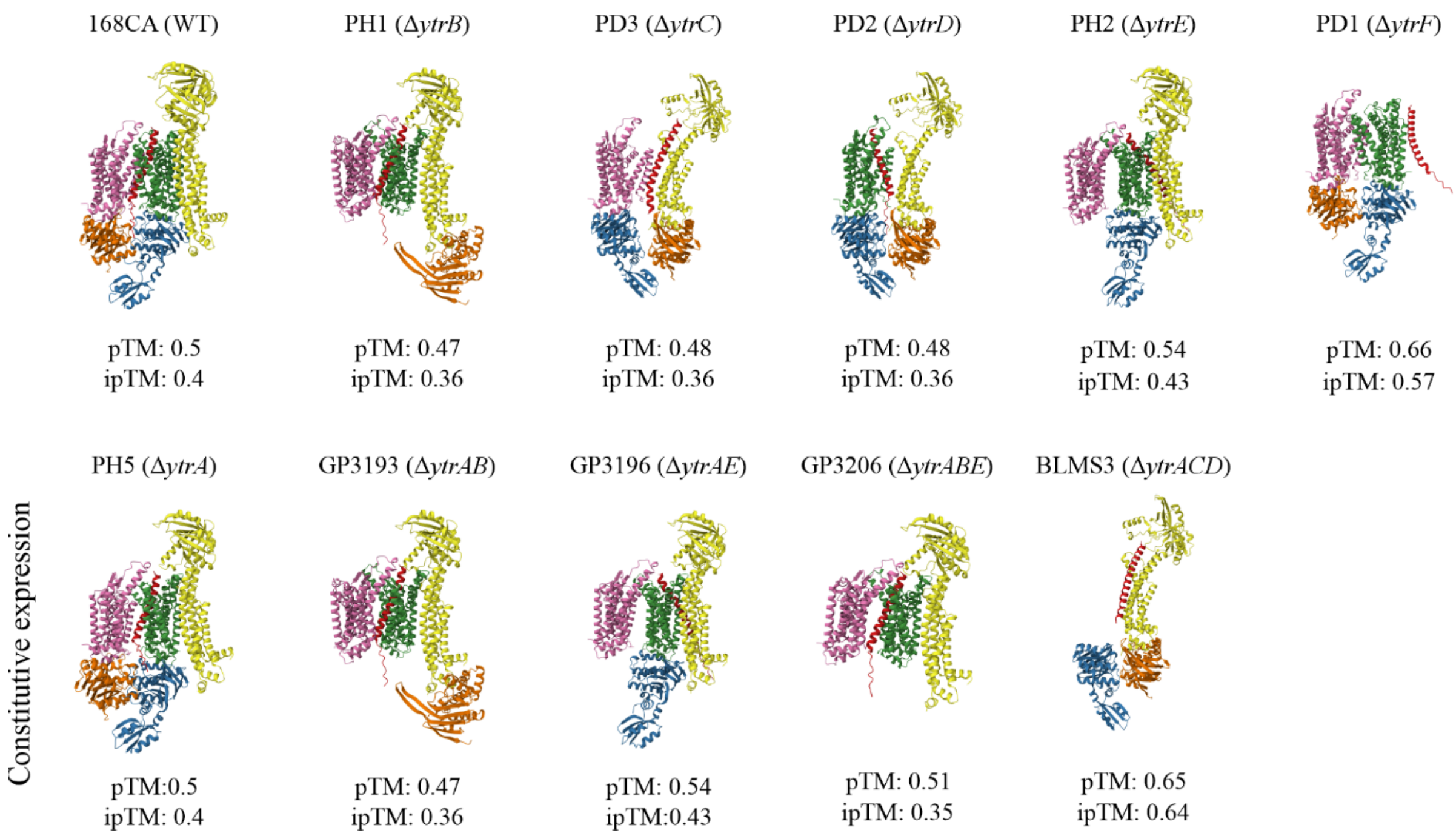

Figure S1: AlphaFold3 structural predictions of transporter structure in the different deletion mutants used in this study assuming a single transporter complex. Colors coding: blue: YtrB, green: YtrC, pink: YtrD, orange: YtrE, yellow: YtrF, red: YtrG. pTM values indicate confidence in the overall 3D structure on a scale from 0 to 1 (>0.7: high confidence, likely correct overall fold, 0.5–0.7: medium confidence, <0.5: low confidence, overall fold may be unreliable). ipTM values indicate confidence in the interactions between individual molecules on a scale from 0 to 1 ((>0.7: high confidence, likely correct interactions, 0.5–0.7: medium confidence, <0.5: low confidence, interactions may be unreliable). Note that structural representations are for visualization of the expressed subunits in the discussed scenarios only and that the stoichiometry and structure of the transporter variants in the different mutant strains have not been experimentally confirmed.

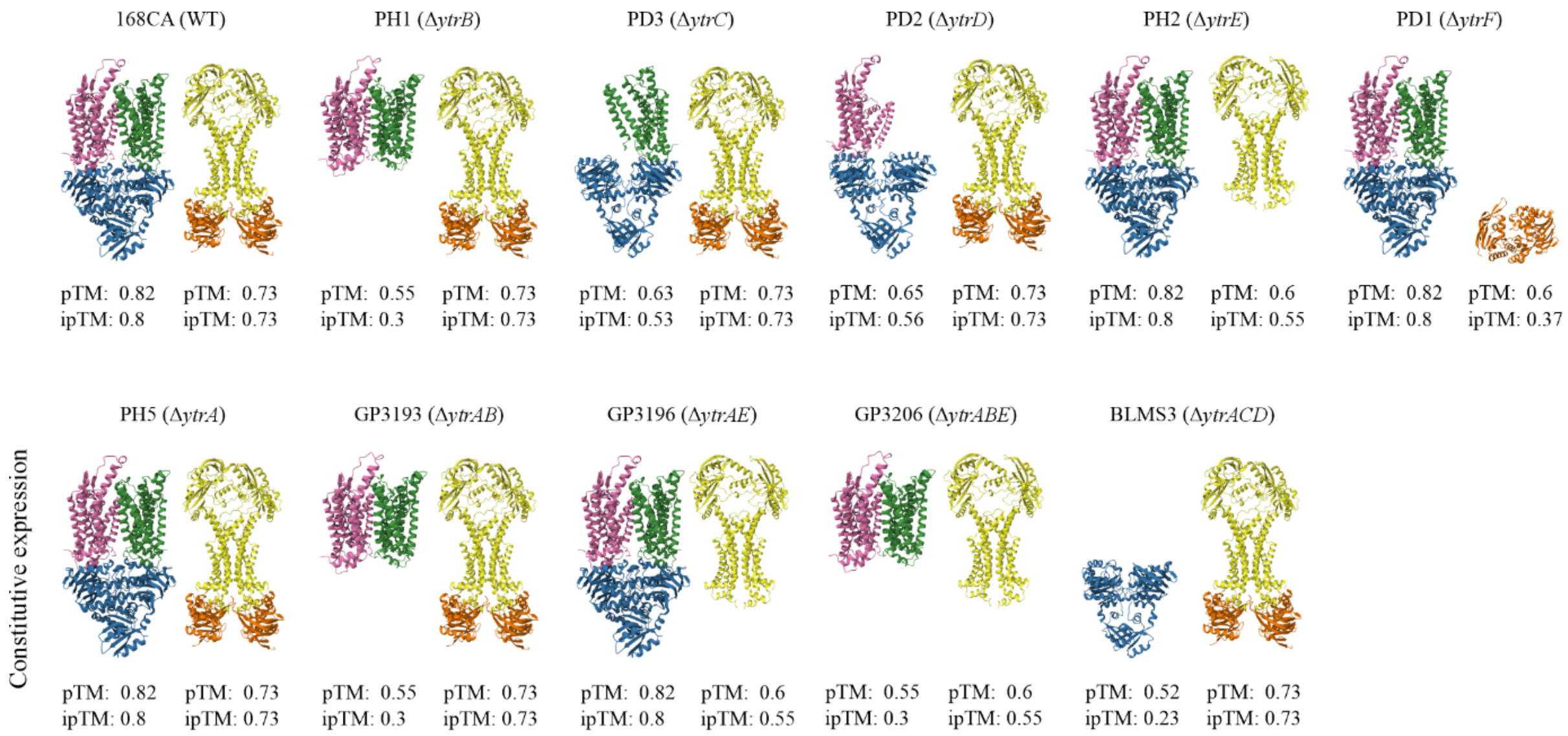

Figure S2: AlphaFold3 structural predictions of transporter structure in the different deletion mutants used in this study assuming two distinct transporters, $YtrB_2CD$ and $YtrE_2F_2$. Each structure is accompanied by its corresponding pTM and ipTM values. Note that YtrG was left out from all structure predictions due to uncertainty regarding how many YtrG proteins to include when assuming two distinct transporters. Colors coding: blue: YtrB, green: YtrC, pink: YtrD, orange: YtrE, yellow: YtrF. pTM values indicate confidence in the overall 3D structure on a scale from 0 to 1 (>0.7: high confidence, likely correct overall fold, 0.5–0.7: medium confidence, <0.5: low confidence, overall fold may be unreliable). ipTM values indicate confidence in the interactions between individual molecules on a scale from 0 to 1 ((>0.7: high confidence, likely correct interactions, 0.5–0.7: medium confidence, <0.5: low confidence, interactions may be unreliable). YtrG is not included in these models as Alphafold did not provide conclusive predictions on the number of YtrG subunits for either $YtrB_2CD$ or $YtrE_2F_2$. Note that structural representations are for visualization of the expressed subunits in the discussed scenarios only and that the stoichiometry and structure of the transporter variants in the different mutant strains have not been experimentally confirmed.

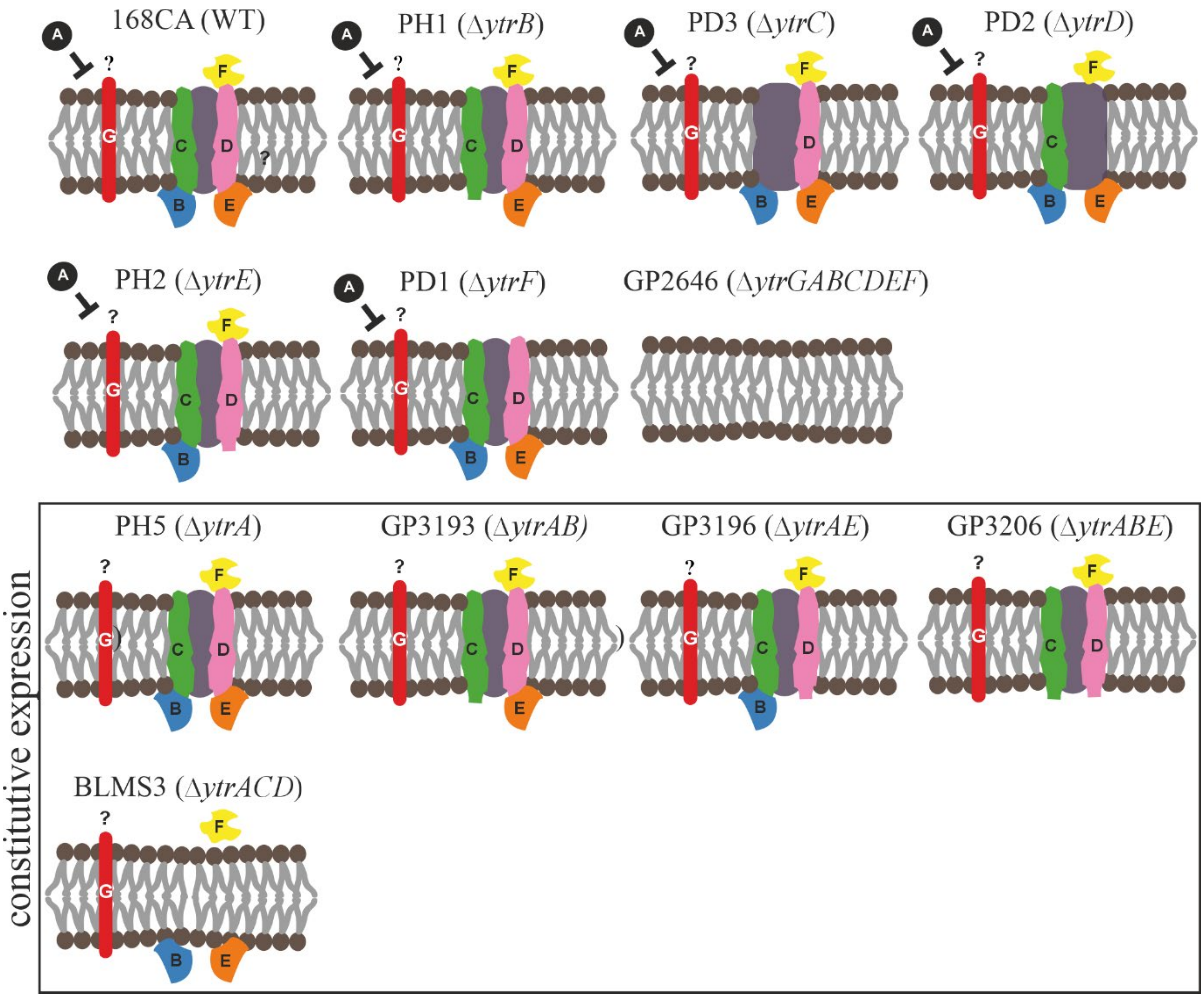

Figure S3: Schematic structure of the putative ABC transporter encoded by the *ytrGABCDEF* operon (WT) and transporter expression in the different deletion mutants used in this study assuming a single transporter complex. Note that deletion of *ytrA* results in constitutive expression of the remaining *ytr* genes (black box). The function of YtrG is not known. Structural predictions classify it as small transmembrane protein[24]. Note that topological representations are for visualization of the expressed subunits in the discussed scenarios only and that the stoichiometry and interactions of the transporter variants in the different mutant strains have not been experimentally confirmed.

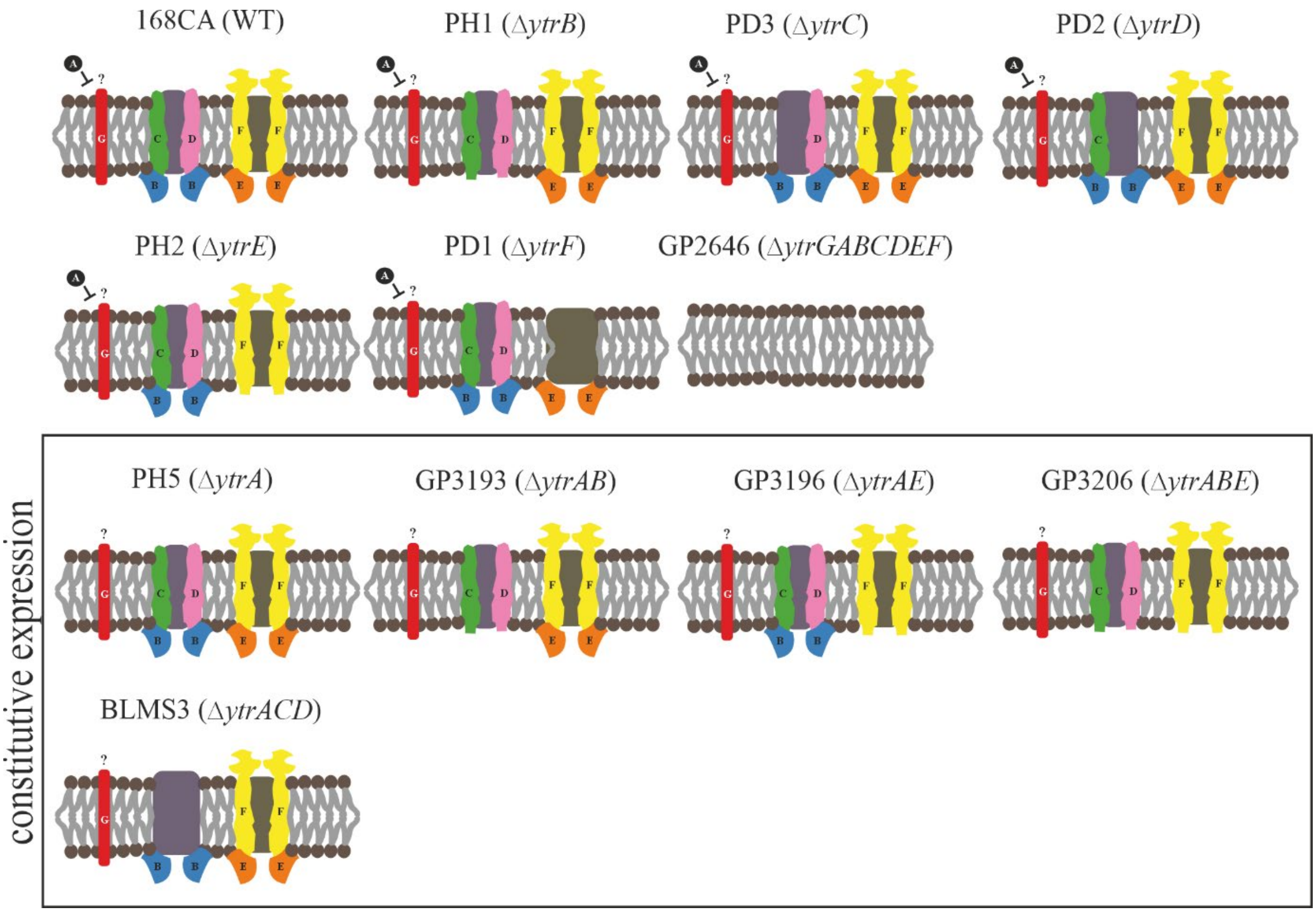

Figure S4: Schematic structure of the putative ABC transporter encoded by the *ytrGABCDEF* operon (WT) and transporter expression in the different deletion mutants used in this study assuming two distinct transporters, $YtrB_2CD$ and $YtrE_2F_2$. Note that deletion of *ytrA* results in constitutive expression of the remaining *ytr* genes (black box). The function of YtrG is not known. Structural predictions classify it as small transmembrane protein[24]. Note that topological representations are for visualization of the expressed subunits in the discussed scenarios only and that the stoichiometry and interactions of the transporter variants in the different mutant strains have not been experimentally confirmed.

A

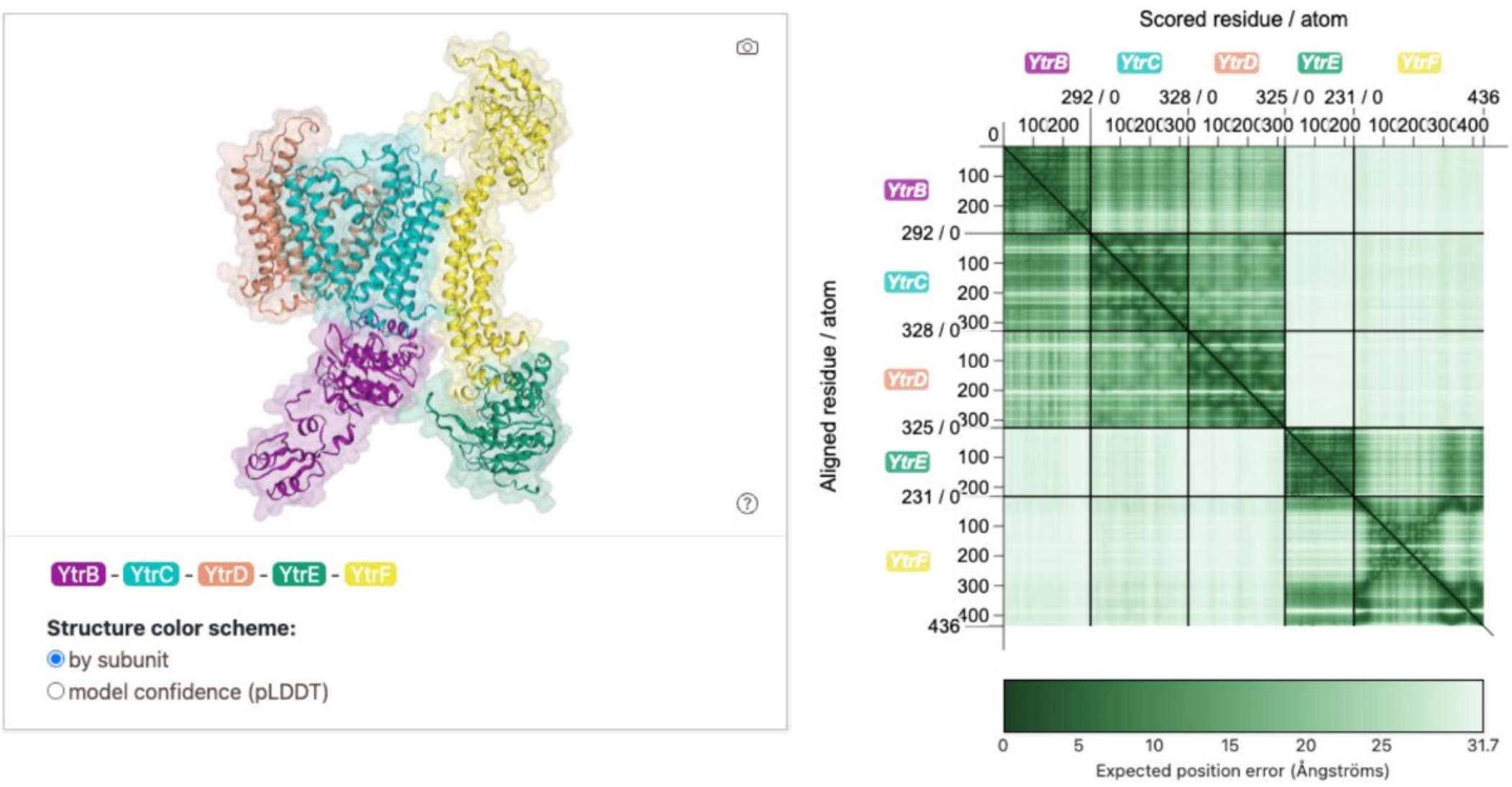

B

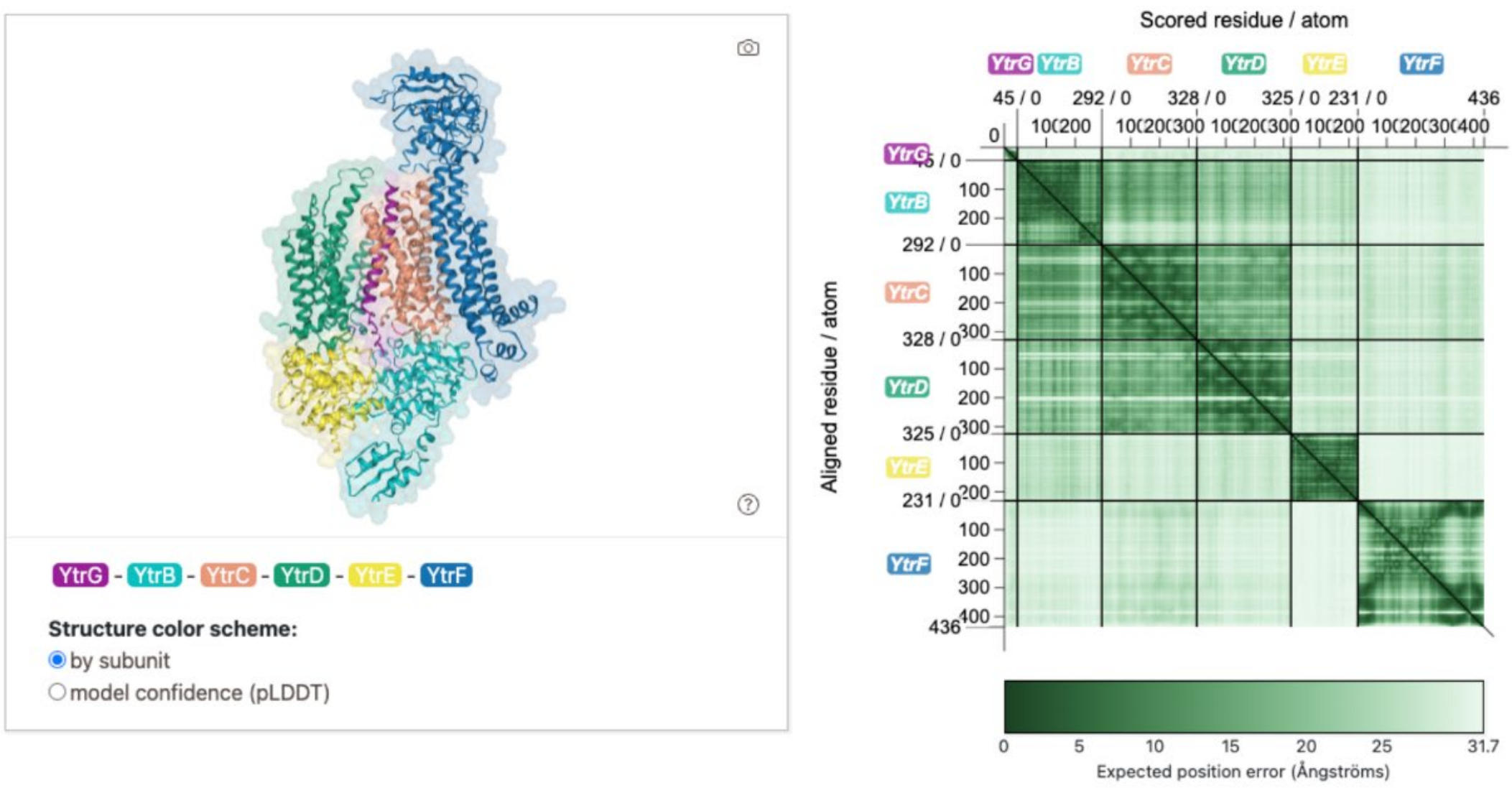

Figure S5: Predicted aligned error (PAE) visualizations of the YtrBCDEF (**A**) and YtrGBCDEF (**B**) transporter topologies. The PAE is a measure for the confidence in the relative positions and orientations of parts of the predicted structures. Lower PAE values (darker green in the PAE heat map) represent more confident predictions. To use the interactive PAE viewer features, these graphs can be reproduced using https://pae-viewer.uni-goettingen.de/[25].

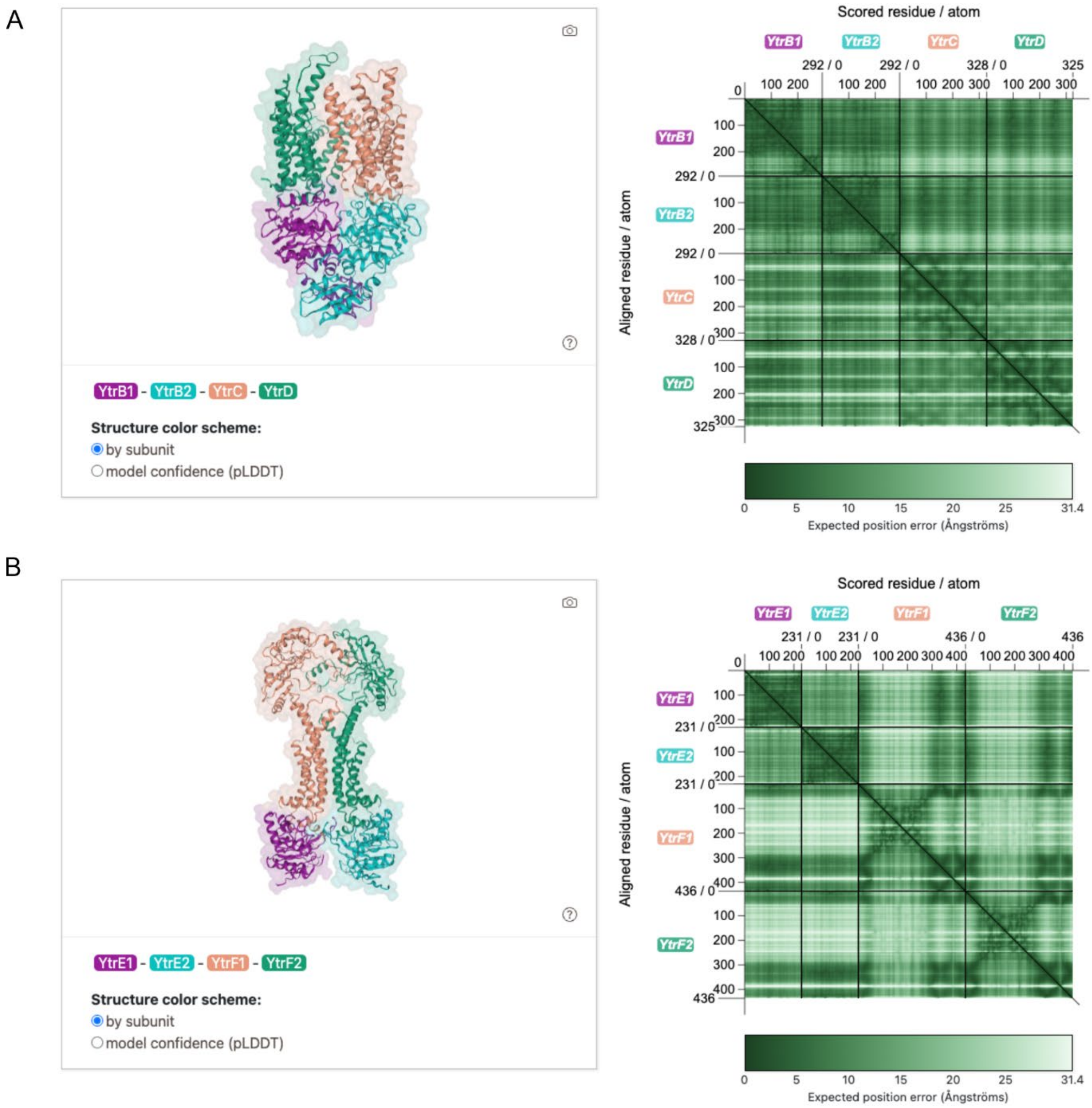

Figure S6: Predicted aligned error (PAE) visualizations of the $YtrB_2CD$ (**A**) and $YtrE_2F_2$ (**B**) transporter topologies. The PAE is a measure for the confidence in the relative positions and orientations of parts of the predicted structures. Lower PAE values (darker green in the PAE heat map) represent more confident predictions. To use the interactive PAE viewer features, these graphs can be reproduced using https://pae-viewer.uni-goettingen.de/[25].

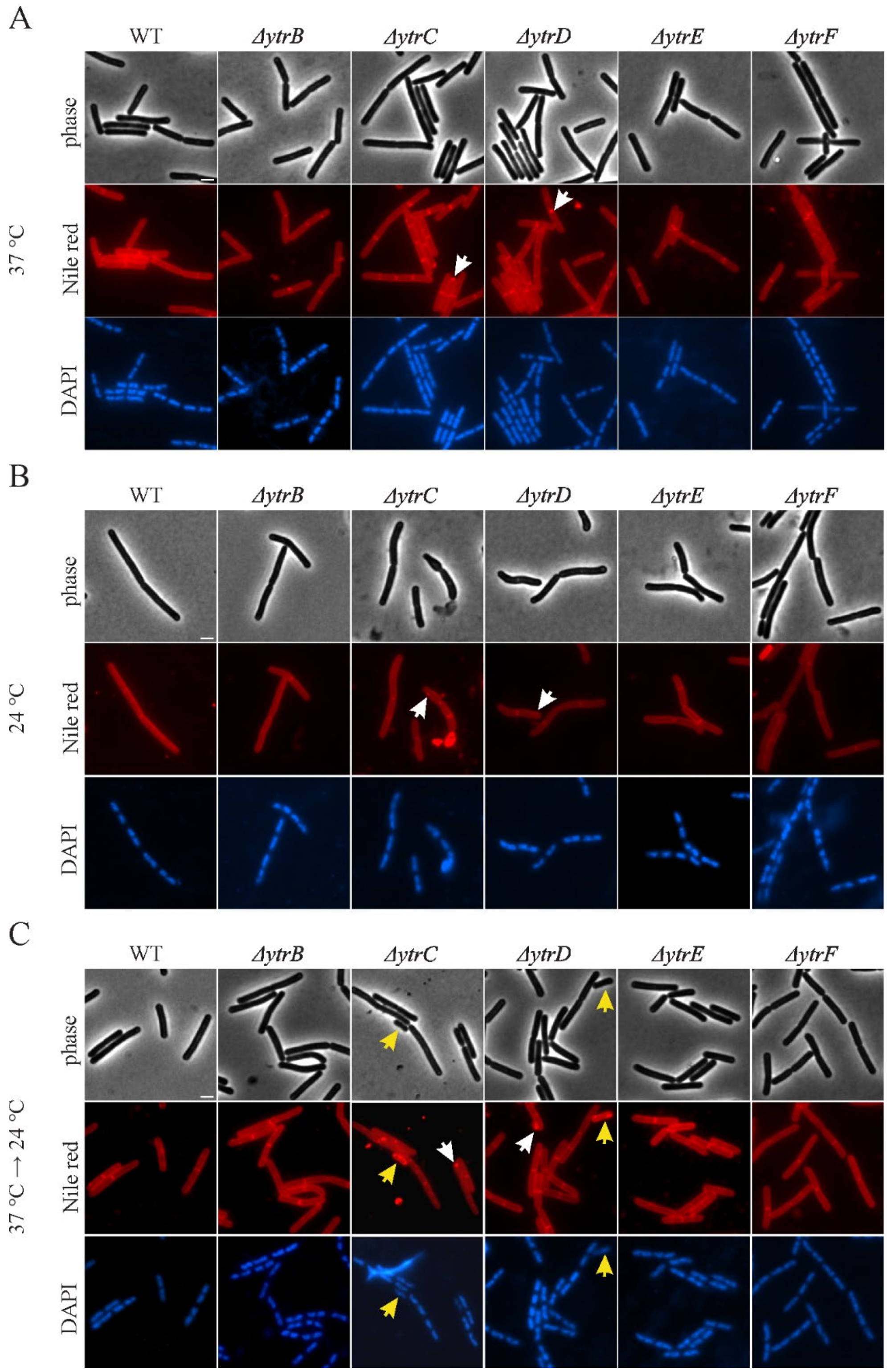

Figure S7: Bacterial cytological profiling (BCP) of WT, Δ*ytrB*, Δ*ytrC*, Δ*ytrD*, Δ*ytrE,* and Δ*ytrF* cultures grown at constant 37 °C (**A**), constant 24 °C (**B**), or shifted from 37 °C to 24 °C after dilution of overnight cultures (**C**). Membranes were stained with Nile red and DNA with DAPI. White arrows indicate membrane foci. Yellow arrows indicate small cells. Scale bars 2 µm.

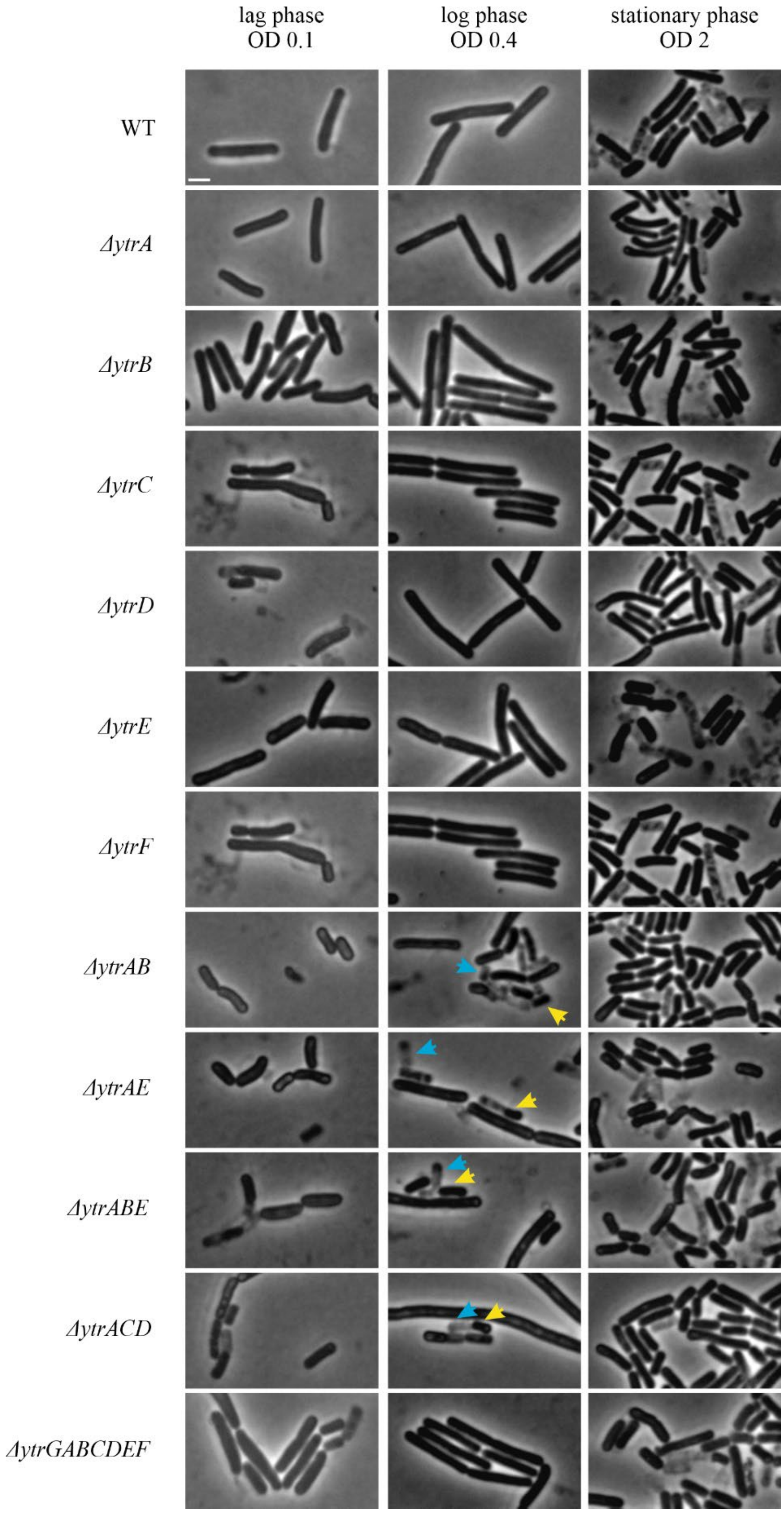

Figure S8: Phase contrast microscopy of *B. subtilis* 168CA and *ytr* deletion mutants grown at 37 °C. Yellow arrows indicate small cells. Blue arrows indicate lysing/lysed small cells. Scale bar 2 µm.

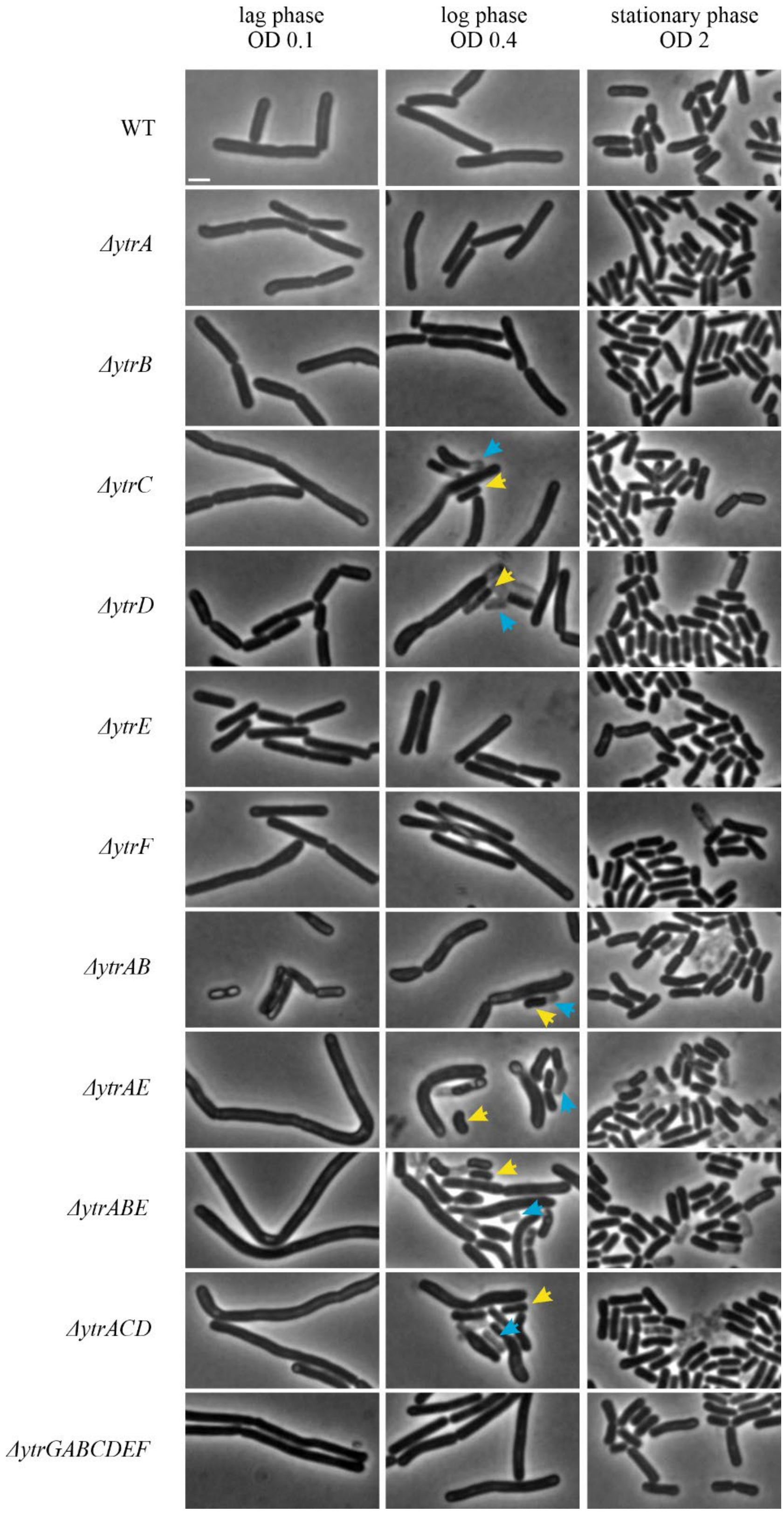

Figure S9: Phase contrast microscopy of *B. subtilis* 168CA and *ytr* deletion mutants grown at 24 °C. Yellow arrows indicate small cells. Blue arrows indicate lysing/lysed small cells. Scale bar 2 µm.

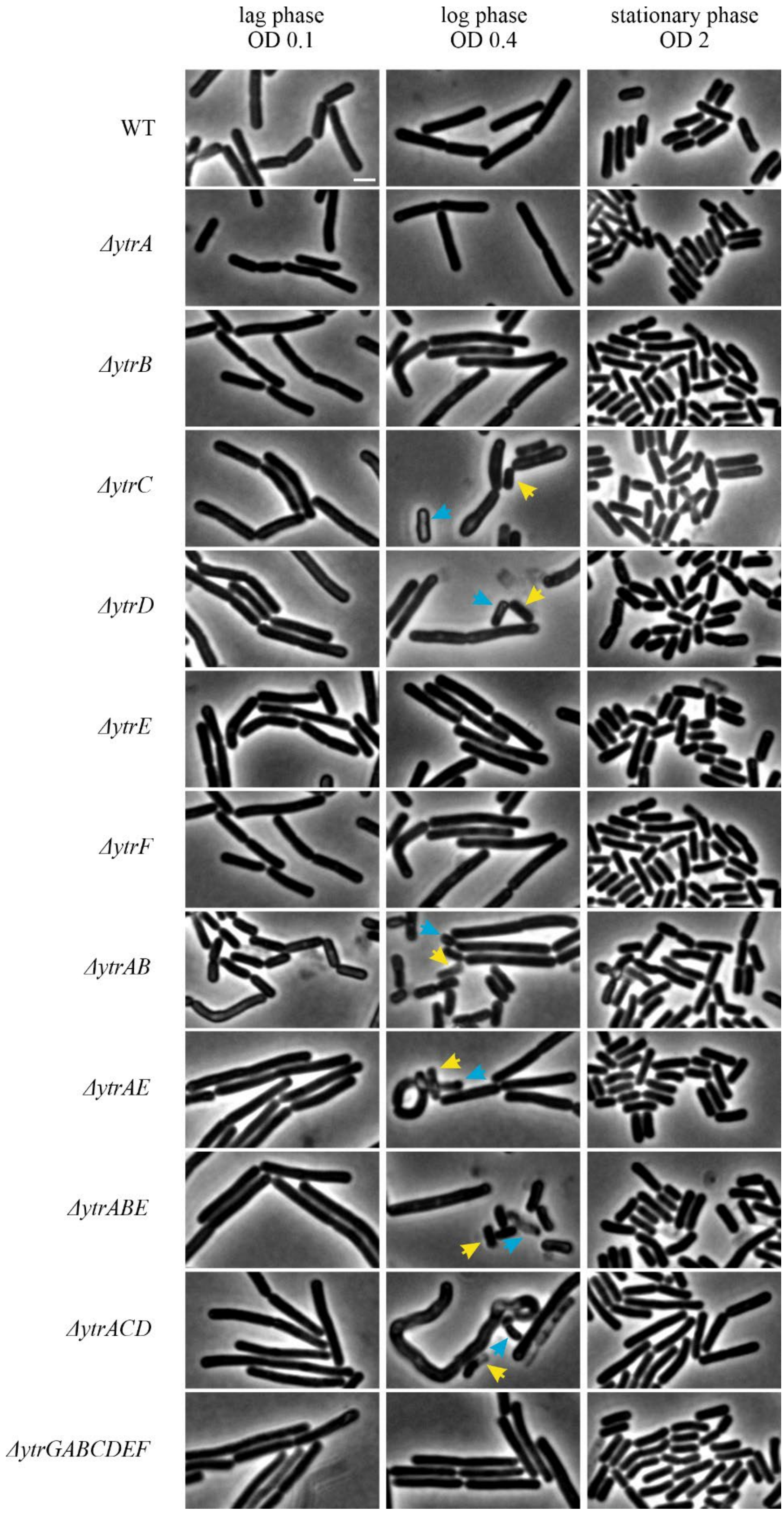

Figure S10: Phase contrast microscopy of *B. subtilis* 168CA and *ytr* deletion mutants shifted from 37 to 24 °C after dilution of overnight cultures. Yellow arrows indicate small cells. Blue arrows indicate lysing/lysed small cells. Scale bar 2 μm.

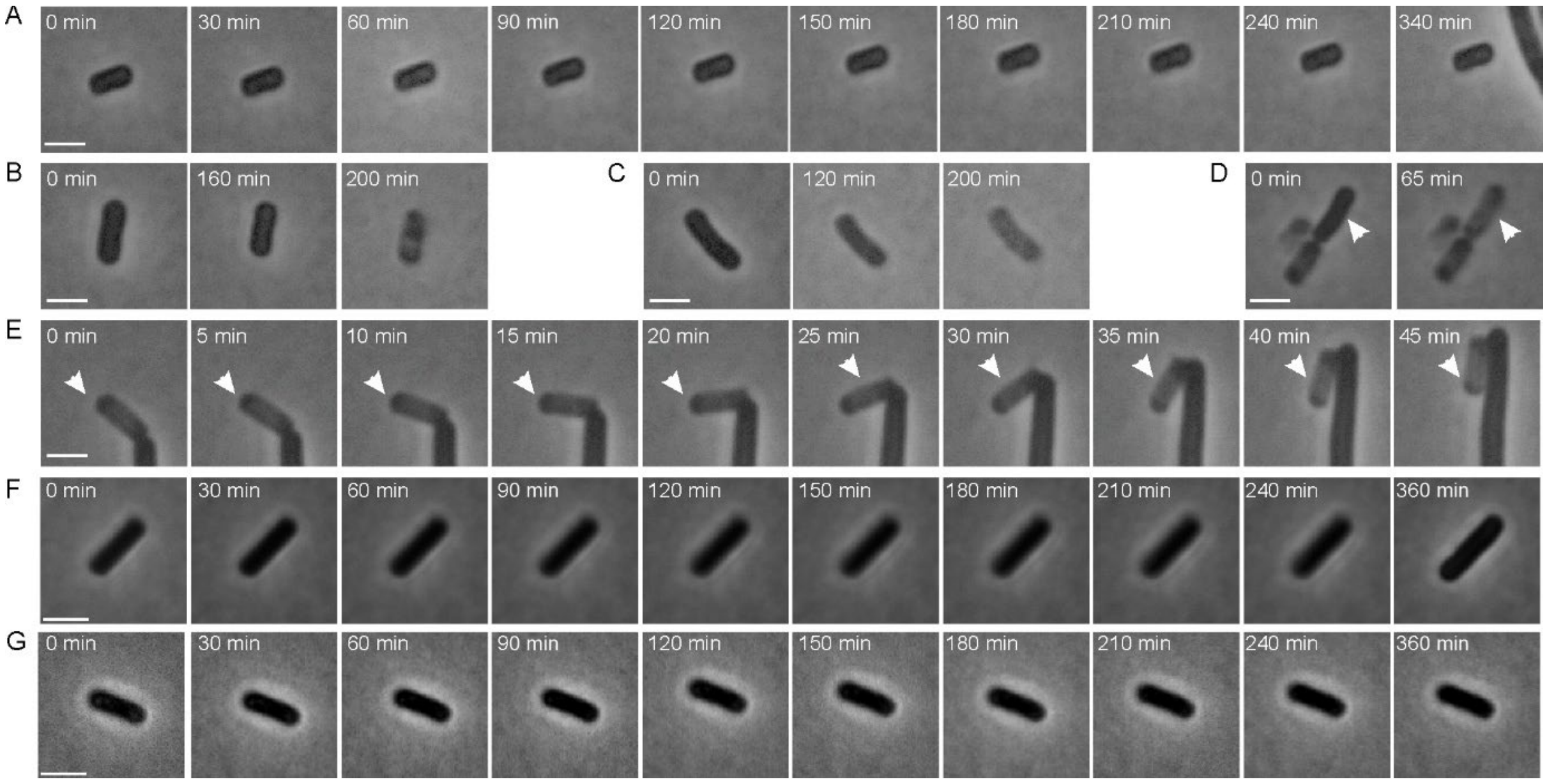

Figure S11: Exemplary timelapse images of small cells. Individual frames were selected from 5-hour timelapse videos. (**A**) Small cells do not grow. (**B-D**) Small cells eventually lyse. (**E**) Small cells derive from actively growing siblings. (**F-G**) Small cells in Δ*ytrA* are not prone to cell lysis. Example images were taken from *B. subtilis* Δ*ytrABE* (A-C), Δ*ytrAE* (D), Δ*ytrGABCDEF* (E), and Δ*ytrA* (F-G). Scale bar 2 µm. Full timelapse videos of *B. subtilis* wild type (168CA), Δ*ytrA* (PH5), Δ*ytrAB* (GP3193), Δ*ytrAE* (GP3196), Δ*ytrABE* (GP3206), Δ*ytrACD* (BLMS3), Δ*ytrGABCDEF* (GP2646), Δ*ytrC* (PD3), and Δ*ytrD* (PD2) can be found as **Video S1-9**.

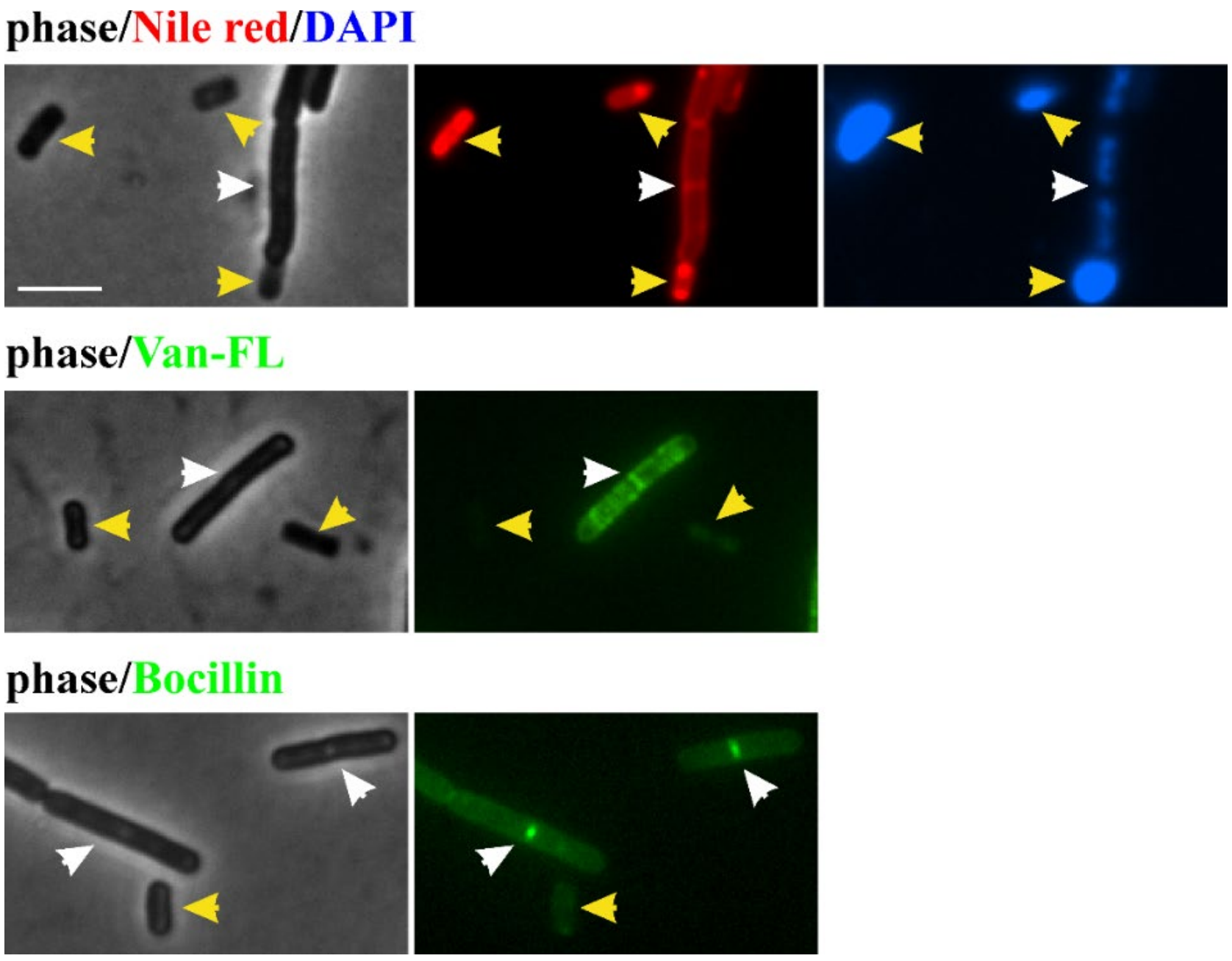

Figure S12: Different fluorescent stainings of small cells and normal cells. Normal cells are indicated with white arrows, small cells with yellow arrows. Images show GP3193 (Δ*ytrAB*) cells grown at 37 °C. Scale bar 2 µm.

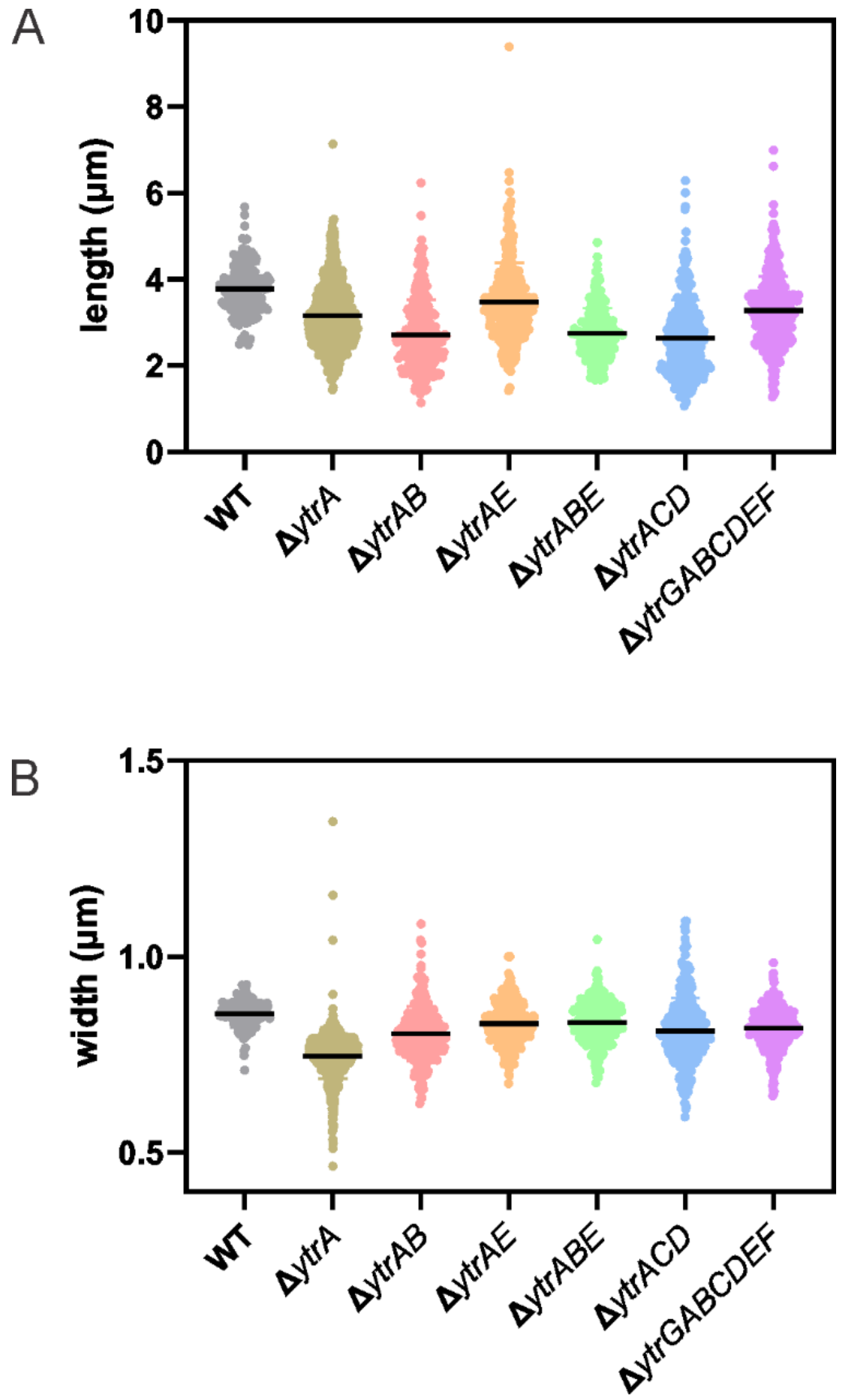

Figure S13: Scatter plots of cell length (**A**) and width (**B**) in different *ytr* deletion mutants.

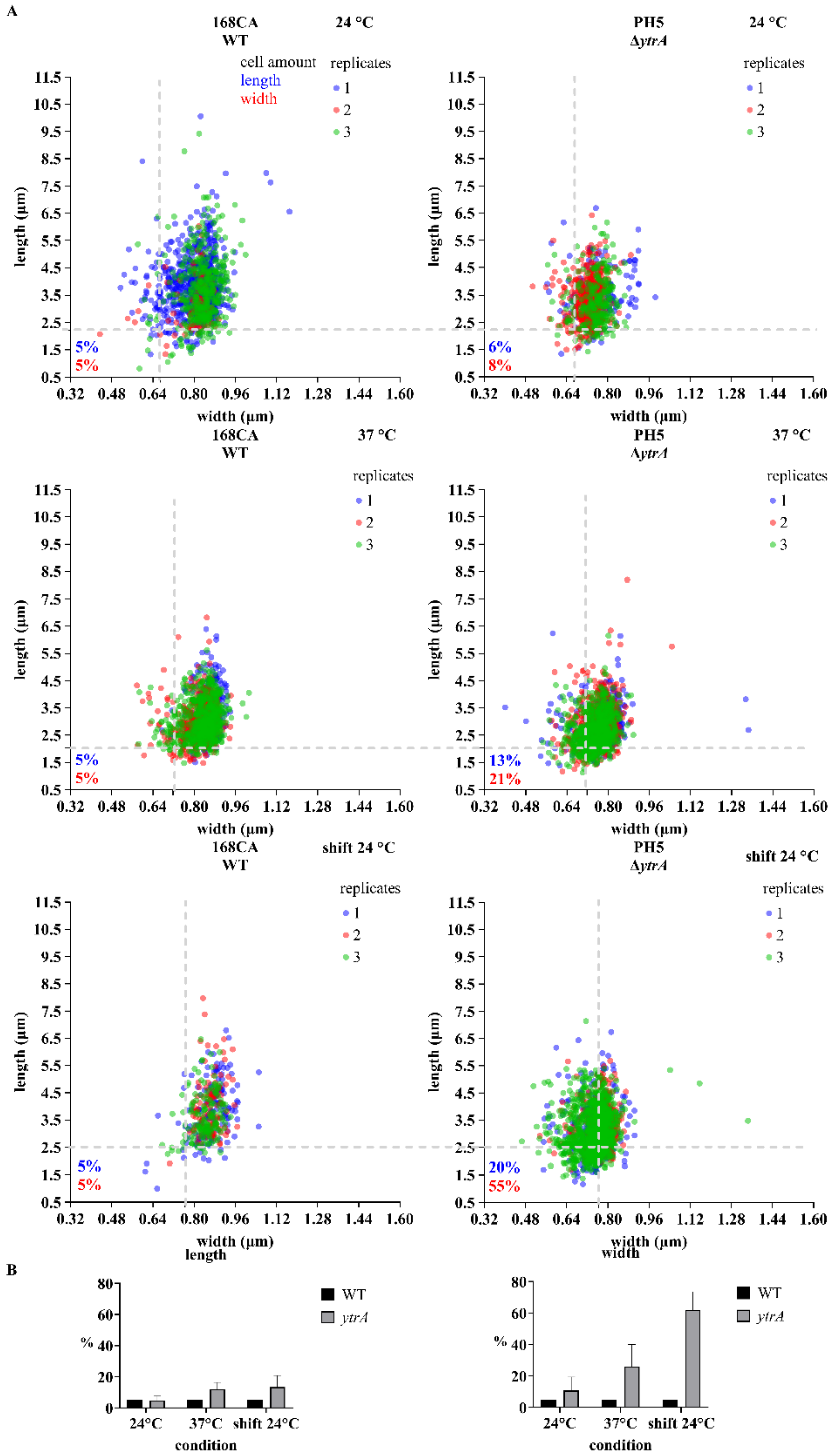

Figure S14: Quantification of cell length and width of *B. subtilis* 168CA (WT) and PH5 (Δ*ytrA*) at different temperature conditions. (**A**) Distribution plots. Blue, red, and green data points represent three biological replicates. Dotted lines represent cutoff values for the 95th quantile for the WT (95% of the WT cell population falling above the cutoff in length and width, respectively). Percentages show the proportion of cells outside of the 5% WT cutoffs for length (red) and width (blue) calculated for pooled datasets. (**B**) Bar charts summarizing the proportions of cells outside of the 95th WT quantile calculated per replicate (mean with standard deviation).

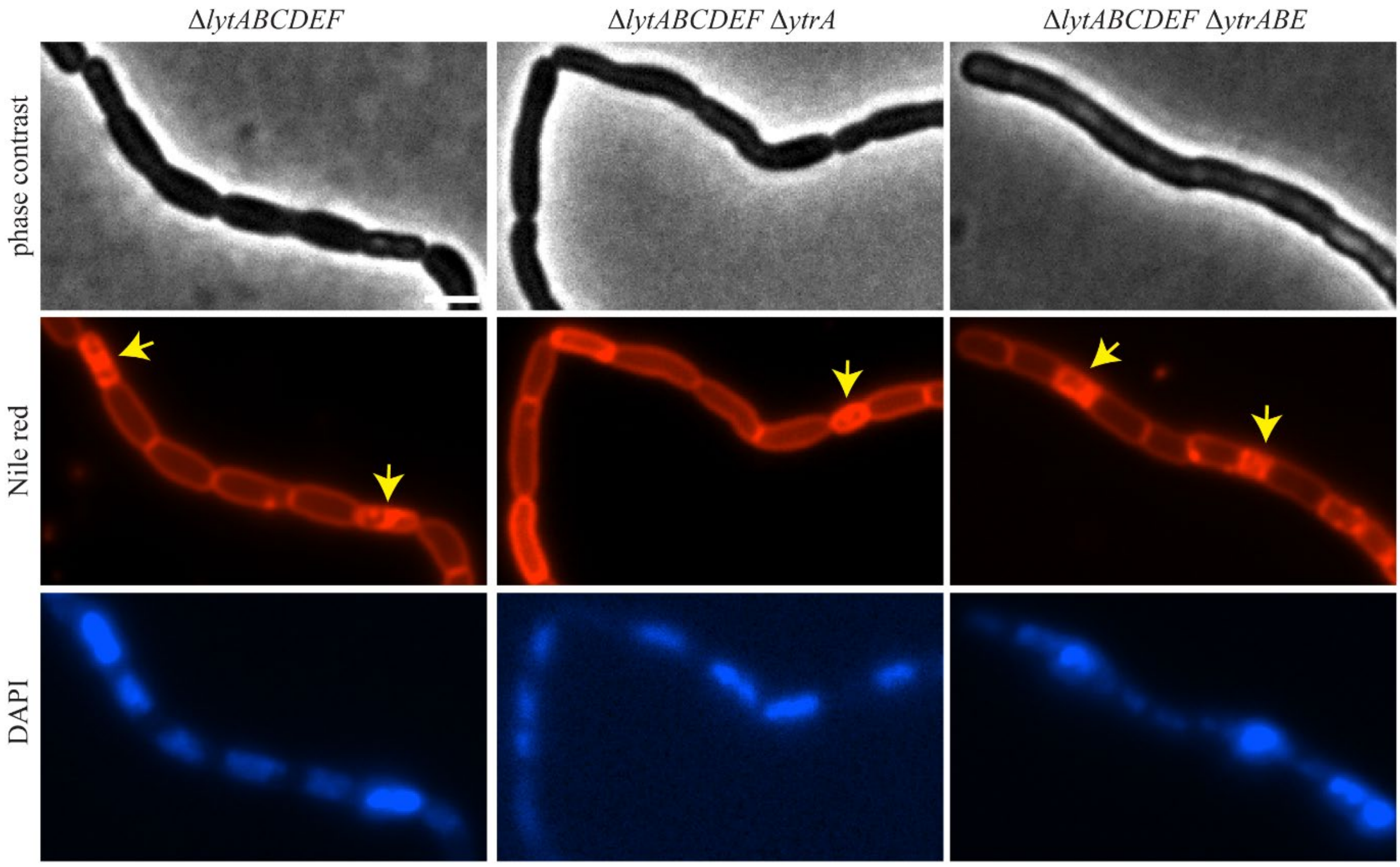

Figure S15: Bacterial cytological profiling of the *B. subtilis* autolysin mutants KS19 (*trpC2 ΔlytABC::kan ΔlytD::tet ΔlytF::spc ΔlytE::cat*), AS2 (*trpC2 ΔlytABC::kan ΔlytD::tet ΔlytF::spc ΔlytE::cat ΔytrA::ery*), and AS3 (*trpC2 ΔlytABC::kan ΔlytD::tet ΔlytF::spc ΔlytE::cat ΔytrA::ery ΔytrB ΔytrE*). Cultures were shifted from 37 °C to 24 °C after dilution of overnight cultures. Note that KS19 and derivatives are impaired in autolytic cell separation causing excessive chain formation. Arrows indicate small cells.

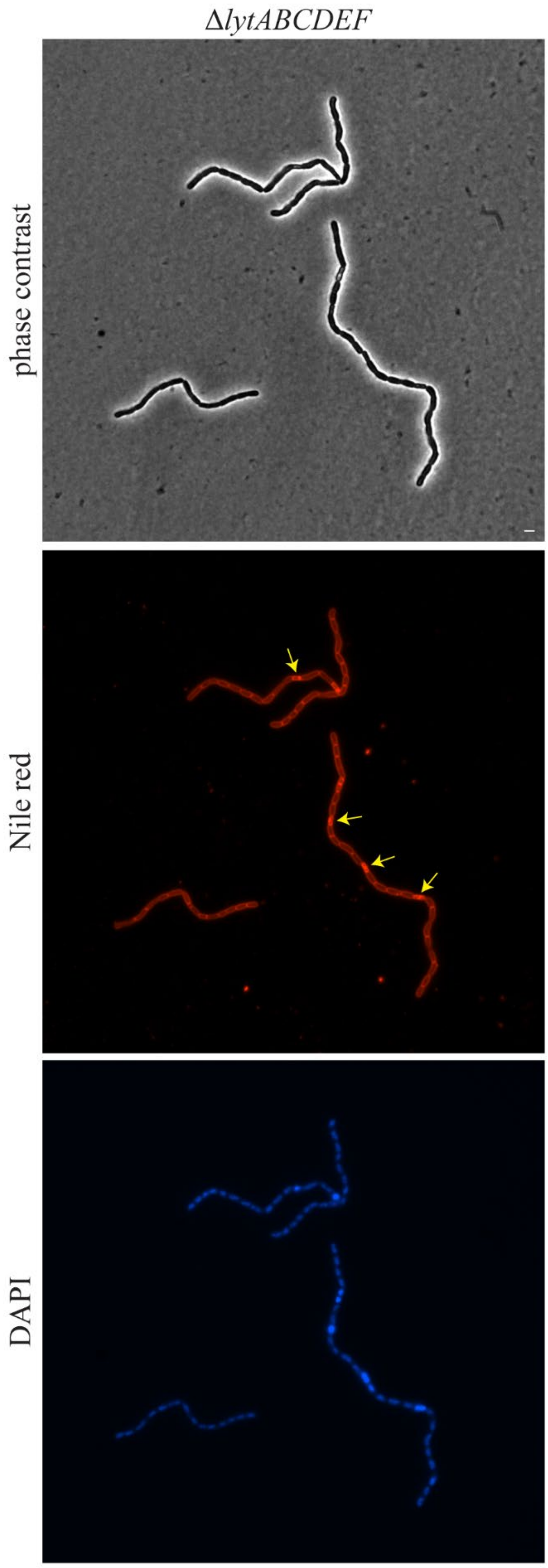

Figure S16: Overview image of bacterial cytological profiling of *B. subtilis* KS19 (*trpC2* Δ*lytABC::kan* Δ*lytD::tet* Δ*lytF::spc* Δ*lytE::cat*) corresponding to **Figure S15**. Note that KS19 is impaired in autolytic cell separation causing excessive chain formation. Arrows indicate small cells.

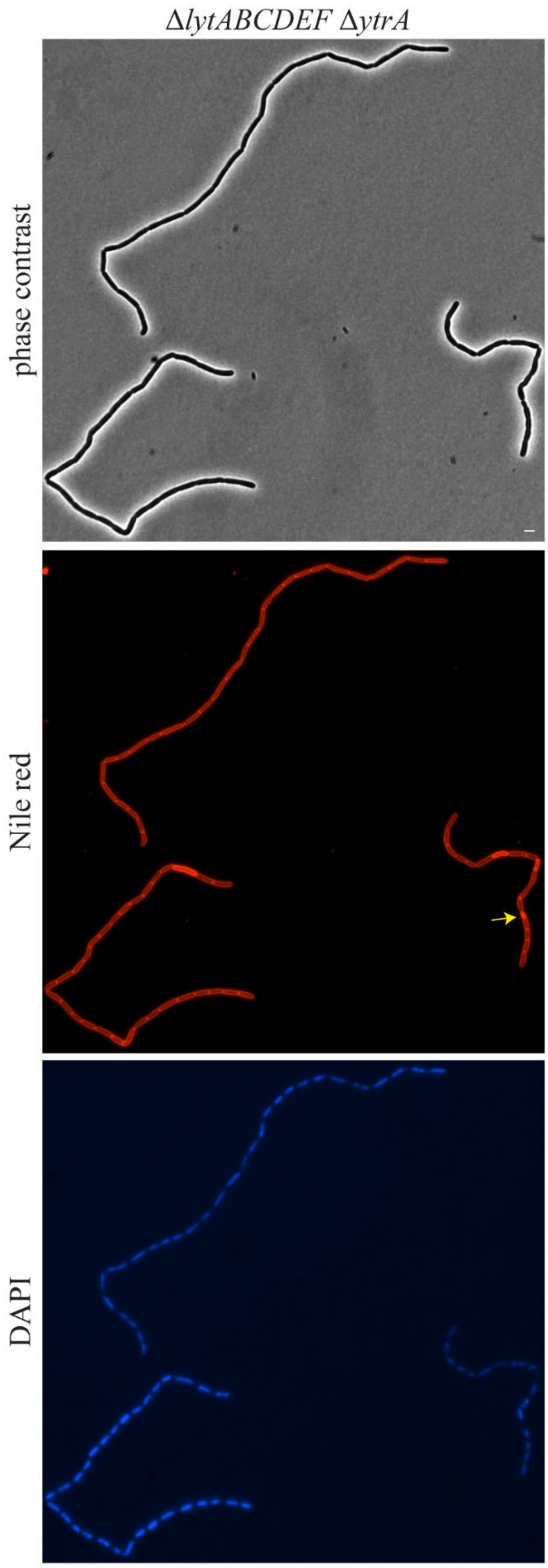

Figure S17: Overview image of bacterial cytological profiling of *B. subtilis* AS2 (*trpC2 ΔlytABC::kan ΔlytD::tet ΔlytF::spc ΔlytE::cat ΔytrA::ery*) corresponding to **Figure S15**. Note that AS2 is impaired in autolytic cell separation causing excessive chain formation. Arrows indicate small cells.

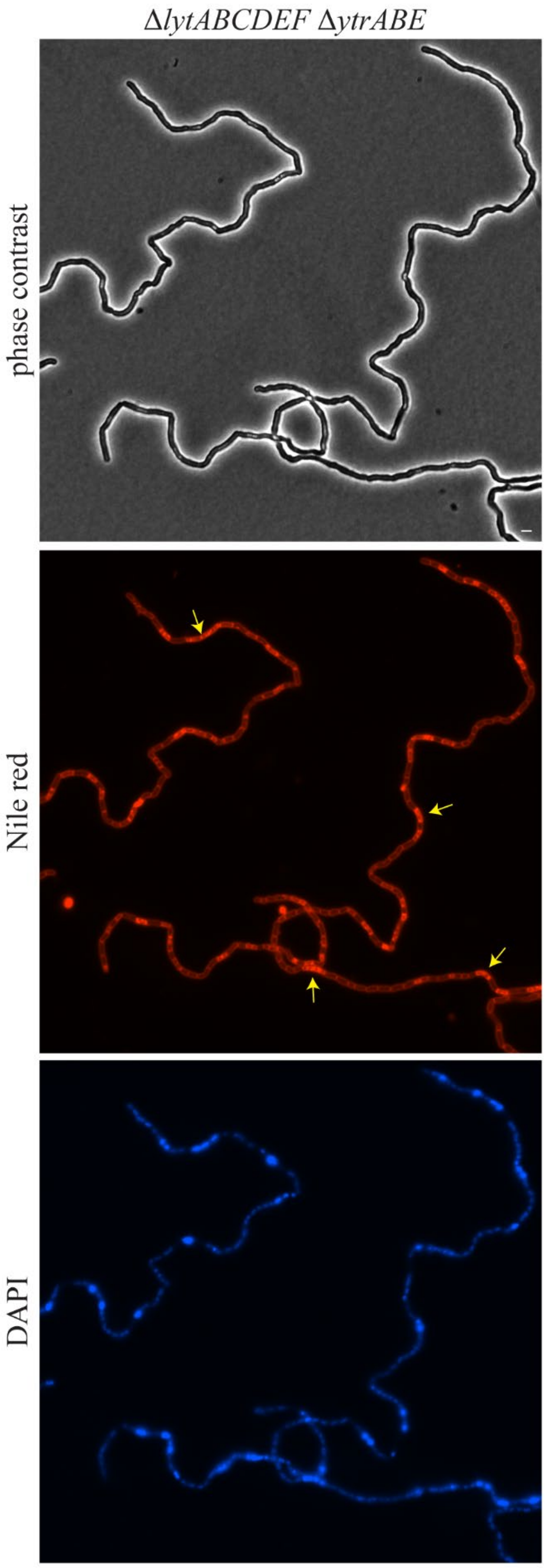

Figure S18: Overview image of bacterial cytological profiling of *B. subtilis* AS3 (*trpC2* Δ*lytABC::kan* Δ*lytD::tet* Δ*lytF::spc* Δ*lytE::cat* Δ*ytrA::ery* Δ*ytrB* Δ*ytrE*) corresponding to **Figure S15**. Note that AS3 is impaired in autolytic cell separation causing excessive chain formation. Arrows indicate small cells.

Video S1: Timelapse microscopy of *B. subtilis* 168 CA (WT). Cultures were adjusted to an OD_{600} of 0.05 prior to spotting on timelapse slides. Images were recorded every 5 min for 6 h. Field of view 134.5 µm.

Video S2: Timelapse microscopy of *B. subtilis* PH5 (Δ*ytrA*). Cultures were adjusted to an OD_{600} of 0.05 prior to spotting on timelapse slides. Images were recorded every 5 min for 6 h. Field of view 134.5 µm.

Video S3: Timelapse microscopy of *B. subtilis* GP3193 (Δ*ytrAB*). Cultures were adjusted to an OD_{600} of 0.05 prior to spotting on timelapse slides. Images were recorded every 5 min for 6 h. Field of view 134.5 µm.

Video S4: Timelapse microscopy of *B. subtilis* GP3196 (Δ*ytrAE*). Cultures were adjusted to an OD_{600} of 0.05 prior to spotting on timelapse slides. Images were recorded every 5 min for 6 h. Field of view 134.5 µm.

Video S5: Timelapse microscopy of *B. subtilis* GP3206 (Δ*ytrABE*). Cultures were adjusted to an OD_{600} of 0.05 prior to spotting on timelapse slides. Images were recorded every 5 min for 6 h. Field of view 134.5 µm.

Video S6: Timelapse microscopy of *B. subtilis* BLMS3 (Δ*ytrACD*). Cultures were adjusted to an OD_{600} of 0.05 prior to spotting on timelapse slides. Images were recorded every 5 min for 6 h. Field of view 134.5 µm.

Video S7: Timelapse microscopy of *B. subtilis* GP2646 (Δ*ytrGABCDEF*). Cultures were adjusted to an OD_{600} of 0.05 prior to spotting on timelapse slides. Images were recorded every 5 min for 6 h. Field of view 134.5 µm.

Video S8: Timelapse microscopy of *B. subtilis* PD3 (Δ*ytrC*). Cultures were adjusted to an OD_{600} of 0.05 prior to spotting on timelapse slides. Images were recorded every 5 min for 6 h. Field of view 134.5 µm.

Video S9: Timelapse microscopy of *B. subtilis* PD2 (Δ*ytrC*). Cultures were adjusted to an OD_{600} of 0.05 prior to spotting on timelapse slides. Images were recorded every 5 min for 6 h. Field of view 134.5 µm.

Video S10: Timelapse microscopy of *B. subtilis* AS3 (Δ*lytABC::kan* Δ*lytD::tet* Δ*lytF::spc* Δ*lytE::cat* Δ*ytrA::ery* Δ*ytrB* Δ*ytrE*). Cultures were adjusted to an OD_{600} of 0.05 prior to spotting on timelapse slides. Images were recorded every 5 min for 6 h. Field of view 134.5 µm. Note that KS19 and derivatives are impaired in autolytic cell separation causing excessive chain formation.

References

(1) Quentin, Y.; Fichant, G.; Denizot, F. Inventory, Assembly and Analysis of *Bacillus subtilis* ABC Transport Systems. *J. Mol. Biol.* **1999**, *287* (3), 467–484. https://doi.org/10.1006/jmbi.1999.2624.

(2) Yoshida, K. I.; Fujita, Y.; Ehrlich, S. D. An Operon for a Putative ATP-Binding Cassette Transport System Involved in Acetoin Utilization of *Bacillus subtilis*. *J. Bacteriol.* **2000**, *182* (19), 5454–5461. https://doi.org/10.1128/JB.182.19.5454-5461.2000.

(3) Salzberg, L. I.; Luo, Y.; Hachmann, A.-B.; Mascher, T.; Helmann, J. D. The *Bacillus subtilis* GntR Family Repressor YtrA Responds to Cell Wall Antibiotics. *J. Bacteriol.* **2011**, *193* (20), 5793–5801. https://doi.org/10.1128/JB.05862-11.

(4) Benda, M.; Schulz, L. M.; Stülke, J.; Rismondo, J. Influence of the ABC Transporter YtrBCDEF of *Bacillus subtilis* on Competence, Biofilm Formation and Cell Wall Thickness. *Front. Microbiol.* **2021**, *12*, 587035. https://doi.org/10.3389/fmicb.2021.587035.

(5) Mahendran, A.; Orlando, B. J. Genome Wide Structural Prediction of ABC Transporter Systems in *Bacillus subtilis*. *Front. Microbiol.* **2024**, *15*, 1469915. https://doi.org/10.3389/fmicb.2024.1469915.

(6) Yu, P.; Krah, B. S.; Orlando, M. A.; Subramanian, S.; Orlando, B. J. Structural Analysis of a Gram-Positive Type VII ABC Transporter Induced by Cell Wall-Targeting Antibiotics. *Structure* **2026**, *34* (1), 145-156.e4. https://doi.org/10.1016/j.str.2025.10.004.

(7) Nicolas, P.; Mäder, U.; Dervyn, E.; Rochat, T.; Leduc, A.; Pigeonneau, N.; Bidnenko, E.; Marchadier, E.; Hoebeke, M.; Aymerich, S.; Becher, D.; Bisicchia, P.; Botella, E.; Delumeau, O.; Doherty, G.; Denham, E. L.; Fogg, M. J.; Fromion, V.; Goelzer, A.; Hansen, A.; Härtig, E.; Harwood, C. R.; Homuth, G.; Jarmer, H.; Jules, M.; Klipp, E.; Le Chat, L.; Lecointe, F.; Lewis, P.; Liebermeister, W.; March, A.; Mars, R. A. T.; Nannapaneni, P.; Noone, D.; Pohl, S.; Rinn, B.; Rügheimer, F.; Sappa, P. K.; Samson, F.; Schaffer, M.; Schwikowski, B.; Steil, L.; Stülke, J.; Wiegert, T.; Devine, K. M.; Wilkinson, A. J.; van Dijl, J. M.; Hecker, M.; Völker, U.; Bessières, P.; Noirot, P. Condition-Dependent Transcriptome Reveals High-Level Regulatory Architecture in *Bacillus subtilis*. *Science* **2012**, *335* (6072), 1103–1106. https://doi.org/10.1126/science.1206848.

(8) Beckering, C. L.; Steil, L.; Weber, M. H. W.; Völker, U.; Marahiel, M. A. Genomewide Transcriptional Analysis of the Cold Shock Response in *Bacillus subtilis*. *J. Bacteriol.* **2002**, *184* (22), 6395–6402. https://doi.org/10.1128/JB.184.22.6395-6402.2002.

(9) Koo, B.-M.; Kritikos, G.; Farelli, J. D.; Todor, H.; Tong, K.; Kimsey, H.; Wapinski, I.; Galardini, M.; Cabal, A.; Peters, J. M.; Hachmann, A.-B.; Rudner, D. Z.; Allen, K. N.; Typas, A.; Gross, C. A. Construction and Analysis of Two Genome-Scale Deletion Libraries for *Bacillus subtilis*. *Cell Syst.* **2017**, *4* (3), 291-305.e7. https://doi.org/10.1016/j.cels.2016.12.013.

(10) Baruah, L.; Sidarta, M.; Hammer úr Skúoy, P.; Johnsson, O.; Frisk, E.; Didelot, P.; Arshad, A.; Wenzel, M. Contribution of the *Bacillus subtilis YtrGABCDEF* Operon to Antibiotic Stress Adaptation. *Microbiol. Spectr.* **2026**, *14* (1), e0269425. https://doi.org/10.1128/spectrum.02694-25.

(11) Cao, M.; Wang, T.; Ye, R.; Helmann, J. D. Antibiotics That Inhibit Cell Wall Biosynthesis Induce Expression of the *Bacillus subtilis* Sigma(W) and Sigma(M) Regulons. *Mol. Microbiol.* **2002**, *45* (5), 1267–1276. https://doi.org/10.1046/j.1365-2958.2002.03050.x.

(12) Schneider, T.; Kruse, T.; Wimmer, R.; Wiedemann, I.; Sass, V.; Pag, U.; Jansen, A.; Nielsen, A. K.; Mygind, P. H.; Raventós, D. S.; Neve, S.; Ravn, B.; Bonvin, A. M. J. J.;

De Maria, L.; Andersen, A. S.; Gammelgaard, L. K.; Sahl, H.-G.; Kristensen, H.-H. Plectasin, a Fungal Defensin, Targets the Bacterial Cell Wall Precursor Lipid II. *Science (80-.).* **2010**, *328* (5982), 1168–1172. https://doi.org/10.1126/science.1185723.
(13) Mascher, T.; Margulis, N. G.; Wang, T.; Ye, R. W.; Helmann, J. D. Cell Wall Stress Responses in *Bacillus subtilis*: The Regulatory Network of the Bacitracin Stimulon. *Mol. Microbiol.* **2003**, *50* (5), 1591–1604.
(14) Bandow, J. E.; Brötz, H.; Leichert, L. I. O.; Labischinski, H.; Hecker, M.; Brotz, H.; Leichert, L. I. O.; Labischinski, H.; Hecker, M. Proteomic Approach to Understanding Antibiotic Action. *Antimicrob. Agents Chemother.* **2003**, *47* (3), 948–955. https://doi.org/10.1128/AAC.47.3.948.
(15) Hutter, B.; Fischer, C.; Jacobi, A.; Schaab, C.; Loferer, H. Panel of *Bacillus Subtilis* Reporter Strains Indicative of Various Modes of Action. *Antimicrob. Agents Chemother.* **2004**, *48* (7), 2588–2594. https://doi.org/10.1128/AAC.48.7.2588-2594.2004.
(16) Wenzel, M.; Kohl, B.; Münch, D.; Raatschen, N.; Albada, H. B.; Hamoen, L.; Metzler-Nolte, N.; Sahl, H. G.; Bandow, J. E. Proteomic Response of *Bacillus subtilis* to Lantibiotics Reflects Differences in Interaction with the Cytoplasmic Membrane. *Antimicrob. Agents Chemother.* **2012**, *56* (11), 5749–5757. https://doi.org/10.1128/AAC.01380-12.
(17) Münch, D.; Müller, A.; Schneider, T.; Kohl, B.; Wenzel, M.; Bandow, J. E.; Maffioli, S.; Sosio, M.; Donadio, S.; Wimmer, R.; Sahl, H.-G. G.; Münch, D.; Müller, A.; Schneider, T.; Kohl, B.; Wenzel, M.; Bandow, J. E.; Maffioli, S.; Sosio, M.; Donadio, S.; Wimmer, R.; Sahl, H.-G. G.; Munch, D.; Muller, A.; Schneider, T.; Kohl, B.; Wenzel, M.; Bandow, J. E.; Maffioli, S.; Sosio, M.; Donadio, S.; Wimmer, R.; Sahl, H.-G. G. The Lantibiotic NAI-107 Binds to Bactoprenol-Bound Cell Wall Precursors and Impairs Membrane Functions. *J. Biol. Chem.* **2014**, *289* (17), 12063–12076. https://doi.org/10.1074/jbc.M113.537449.
(18) Wenzel, M.; Chiriac, A. I.; Otto, A.; Zweytick, D.; May, C.; Schumacher, C.; Gust, R.; Albada, H. B.; Penkova, M.; Krämer, U.; Erdmann, R.; Metzler-Nolte, N.; Straus, S. K.; Bremer, E.; Becher, D.; Brötz-Oesterhelt, H.; Sahl, H.-G.; Bandow, J. E. Small Cationic Antimicrobial Peptides Delocalize Peripheral Membrane Proteins. *Proc. Natl. Acad. Sci.* **2014**, *111* (14). https://doi.org/10.1073/pnas.1319900111.
(19) Stepanek, J. J.; Lukezic, T.; Teichert, I.; Petkovic, H.; Bandow, J. E. Dual Mechanism of Action of the Atypical Tetracycline Chelocardin. *Biochim. Biophys. Acta* **2016**, *1864* (6), 645–654. https://doi.org/10.1016/j.bbapap.2016.03.004.
(20) Zhang, Q.; Cornilleau, C.; Müller, R. R.; Meier, D.; Flores, P.; Guérin, C.; Wolf, D.; Fromion, V.; Carballido-Lopez, R.; Mascher, T. Comprehensive and Comparative Transcriptional Profiling of the Cell Wall Stress Response in *Bacillus subtilis*. *bioRxiv* **2023**, 2023.02.03.526509. https://doi.org/10.1101/2023.02.03.526509.
(21) Senges, C. H. R.; Stepanek, J. J.; Wenzel, M.; Raatschen, N.; Ay, Ü.; Märtens, Y.; Prochnow, P.; Vázquez Hernández, M.; Yayci, A.; Schubert, B.; Janzing, N. B. M.; Warmuth, H. L.; Kozik, M.; Bongard, J.; Alumasa, J. N.; Albada, B.; Penkova, M.; Lukežič, T.; Sorto, N. A.; Lorenz, N.; Miller, R.; Zhu, B.; Benda, M.; Stülke, J.; Schäkermann, S.; Leichert, L. I.; Scheinpflug, K.; Brötz-Oesterhelt, H.; Hertweck, C.; Shaw, J. T.; Petković, H.; Brunel, J. M.; Keiler, K. C.; Metzler-Nolte, N.; Bandow, J. E. Comparison of Proteomic Responses as Global Approach to Antibiotic Mechanism of Action Elucidation. *Antimicrob. Agents Chemother.* **2020**, *65* (1), e01373-20. https://doi.org/10.1128/AAC.01373-20.
(22) Spizizen, J. Transformation of Biochemically Deficient Srains of *Bacillus subtilis* by Deoxyribonucleate. *Proc. Natl. Acad. Sci.* **1958**, *44* (10), 1072–1078. https://doi.org/10.1073/pnas.44.10.1072.

(23) Scheinpflug, K.; Wenzel, M.; Krylova, O.; Bandow; E, J.; Dathe, M.; Strahl, H. Antimicrobial Peptide CWFW Kills by Combining Lipid Phase Separation with Autolysis. *Sci. Rep.* **2017**, *7*, 44332. https://doi.org/10.1038/srep44332.
(24) Pedreira, T.; Elfmann, C.; Stülke, J. The Current State of SubtiWiki, the Database for the Model Organism *Bacillus subtilis*. *Nucleic Acids Res.* **2022**, *50* (D1), D875–D882. https://doi.org/10.1093/nar/gkab943.
(25) Elfmann, C.; Stülke, J. PAE Viewer: A Webserver for the Interactive Visualization of the Predicted Aligned Error for Multimer Structure Predictions and Crosslinks. *Nucleic Acids Res.* **2023**, *51* (W1), W404–W410. https://doi.org/10.1093/nar/gkad350.
